# Time-resolved analysis of a denitrifying bacterial community revealed a core microbiome responsible for the anaerobic degradation of quinoline

**DOI:** 10.1038/s41598-017-15122-0

**Published:** 2017-11-07

**Authors:** Yun Wang, Hao Tian, Fei Huang, Wenmin Long, Qianpeng Zhang, Jing Wang, Ying Zhu, Xiaogang Wu, Guanzhou Chen, Liping Zhao, Lars R. Bakken, Åsa Frostegård, Xiaojun Zhang

**Affiliations:** 1 State Key Laboratory of Microbial Metabolism and School of Life Sciences and Biotechnology, Shanghai Jiao Tong University, Shanghai, 200240 P.R. China; 2 Key Laboratory of Biogeography and Bioresource in Arid Land, Xinjiang Institute of Ecology and Geography, Chinese Academy of Sciences, Urumqi, 830011 P.R. China; 3 Faculty of Chemistry, Biotechnology and Food Science, Norwegian University of Life Sciences, Aas, N-1432 Norway

## Abstract

Quinoline is biodegradable under anaerobic conditions, but information about the degradation kinetics and the involved microorganisms is scarce. Here, the dynamics of a quinoline-degrading bacterial consortium were studied in anoxic batch cultures containing nitrate. The cultures removed 83.5% of the quinoline during the first 80 hours, which were dominated by denitrification, and then switched to methanogenesis when the nitrogen oxyanions were depleted. Time-resolved community analysis using pyrosequencing revealed that denitrifiying bacteria belonging to the genus *Thauera* were enriched during the denitrification stage from 12.2% to 38.8% and 50.1% relative abundance in DNA and cDNA libraries, respectively. This suggests that they are key organisms responsible for the initial attack on quinoline. Altogether, 13 different co-abundance groups (CAGs) containing 76 different phylotypes were involved, directly or indirectly, in quinoline degradation. The dynamics of these CAGs show that specific phylotypes were associated with different phases of the degradation. Members of *Rhodococcus* and *Desulfobacterium*, as well as *Rhodocyclaceae*- and *Syntrophobacteraceae*-related phylotypes, utilized initial metabolites of the quinoline, while the resulting smaller molecules were used by secondary fermenters belonging to *Anaerolineae*. The concerted action by the different members of this consortium resulted in an almost complete anaerobic mineralization of the quinoline.

## Introduction

Quinoline and its derivatives are widely used in chemical, pharmaceutical, pesticides and dyeing industries as raw materials and solvents. Owing to their relatively high aqueous solubility and low biodegradability, they have become rife contaminants in groundwater and soil^1–4^.

Aerobic degradation of environmental pollutants is well documented as being a fast and efficient way of dealing with pollutants. However heavily polluted environments are often oxygen deficient^5^, thus restricting the aerobic degradation of quinoline unless oxygen is injected^6^. Anaerobic bioremediation is an attractive technology due to its virtue of cost-effectiveness.

Efficient remediation of quinoline contaminated sites is a challenging task. A number of microorganisms capable of mineralizing quinoline aerobically have been isolated, belonging to genera *Comamonas* ^7^, *Rhodococcus* ^8^ and *Burkholderia* ^9^. The pathways of aerobic quinoline degradation have also been well documented^1^. Studies conducted during the past decades have shown that quinoline is biodegradable under anoxic conditions, albeit slower than in the presence of oxygen^1^. Quinoline biodegradation under anoxic conditions has been demonstrated when nitrate^10,11^ or sulfate are available as electron acceptors^12^, and has also been shown to take place under methanogenic conditions^13^. Present-day knowledge on the biochemical pathway of anaerobic quinoline degradation is limited and based on a few studies^1,4,11^. One reason for this is that the information about which organisms are involved in the degradation is scarce and, secondly, that these organisms largely remain uncultured. To date, *Desulfobacterium indolicum* (DSM 3383) is the only cultured organism that degrades quinoline anaerobically. This organism uses sulfate as electron acceptor during the degradation, and some of the metabolites produced have been identified^12^. Only a few attempts have been made to characterize the dynamics of quinoline degradation and the bacterial consortia involved. In one study, the microbial community from a coking water plant was analyzed when growing on quinoline under anoxic conditions in the presence of nitrate. Denaturing gradient gel electrophoresis (DGGE) followed by sequencing of dominant bands identified the genera *Thauera* and *Azoarcus* as the key functional members^14^. In another study, Zhang *et al*. found that *Alicycliphilus denitrificans* were the dominant contributors in shortcut denitrification quinoline degradation with seed sampled from municipal sewage treatment^15^.

Our laboratory maintained an efficient quinoline-degrading, denitrifying consortium in a continuous flow bioreactor with quinoline as the sole carbon source. Phylogenetic analysis of this consortium showed a high microbial diversity, mainly consisting of organisms belonging to phyla *Proteobacteria*, *Actinobacteria*, and *Bacteroidetes* ^16^. The present study aimed to characterize the quinoline-degrading microbial community and supply more detailed knowledge on the linkage between quinoline biodegradation and denitrification in this consortium. The kinetics of CO_2_, the denitrification products NO_2_^−^, NO, N_2_O and N_2_, as well as methane and ammonia, were measured during anoxic incubation. Changes in the bacterial community composition were analyzed with emphasis on the metabolically active organisms, determined by cDNA sequence analysis of the 16S rRNA transcripts.

## Results

### Biodegradation of quinoline in batch culture

**Figure 1:**
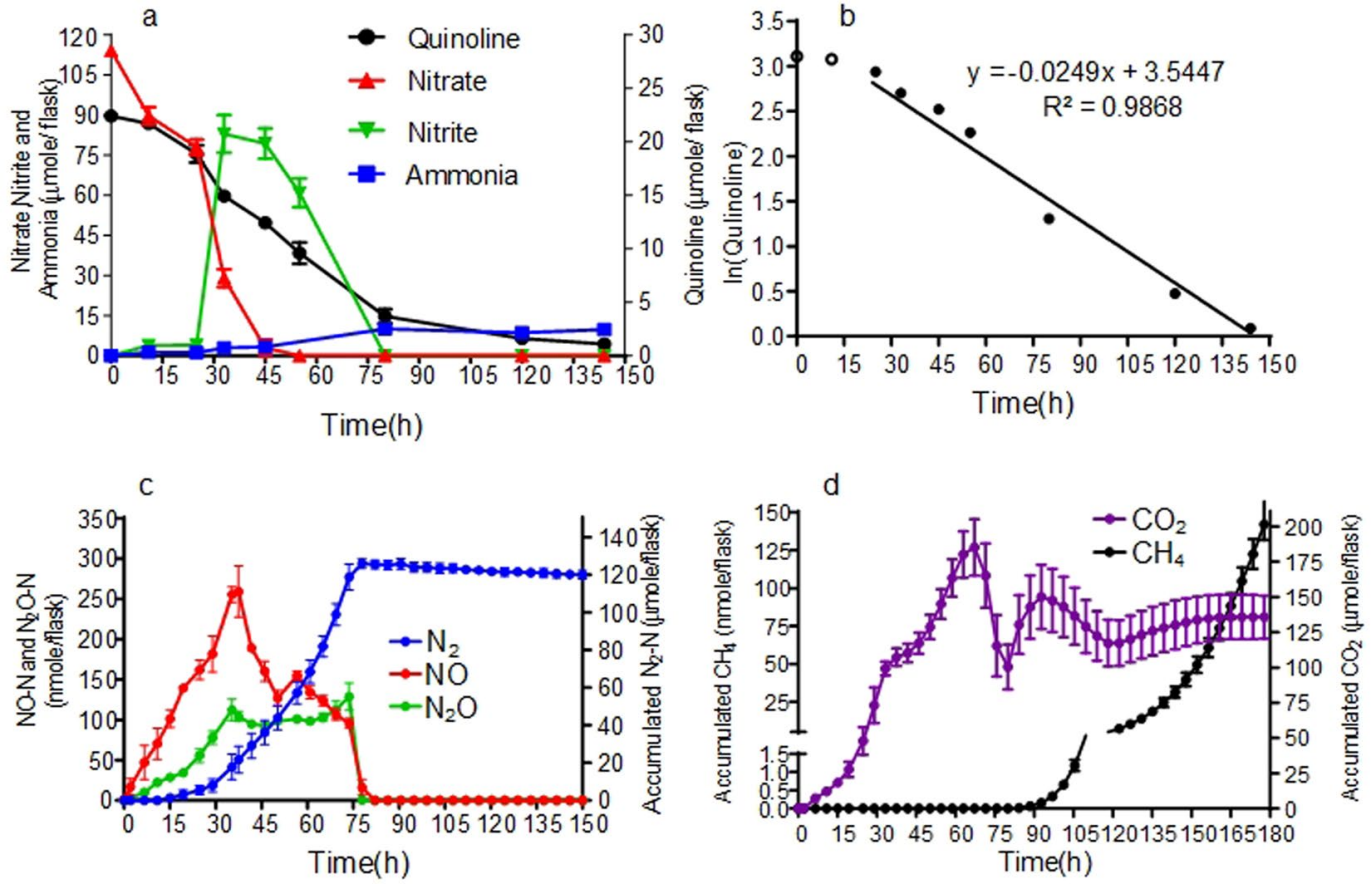
The kinetics of nitrogen species, CO_2_, CH_4_ production and quinoline degradation in batch cultures. Values are shown as mean ± SD. (**a**) Concentration of quinoline, nitrate, nitrite and ammonia. (**b**) shows the logarithm of the quinoline concentrations plotted against time, with a linear regression function for the period from 25 to 144 h. The regression coefficient estimated the first order decay rate (k = 0.025 μmol h^−1^). (**c**) Kinetics of NO, N_2_O, N_2_ during quinoline degradation process. (**d**) Kinetics of CO_2_ and CH_4_ during quinoline degradation process. For N_2_, CO_2_ and CH_4_, which are both end products, accumulated production is shown, i.e. measured concentrations corrected for leakage and sampling losses. For the intermediates, such as NO and N_2_O, the measured values are shown. The declining CO_2_ levels during the fast reduction of nitrite (60–75 h) is probably due to slight increase in pH.

Batch cultures with material from the continuous denitrifying quinoline degradation reactor were set up with medium containing an initial concentration of 100 mg l^−1^ quinoline (23.26 μmol flask^−1^) and 105 μmol flask^−1^ NO_3_^−^. The quinoline degradation was slow initially, but increased during the first 25 h (Fig. 1a). Thereafter, the degradation rate was essentially a first order reaction. Of the initially supplied quinoline, 83.5% was degraded during the first 80 h when denitrification took place. During this phase, degradation of quinoline continued at the same rate as under denitrifying conditions (Fig. 1b). The concentration of ammonia increased along with the biodegradation of quinoline and finally reached a stable level of 10 μmol flask^−1^ (Fig. 1a). When all nitrogen oxides were depleted, methane production started.

During the denitrification phase, nitrate was reduced while nitrite accumulated to a maximum of 82 μmol NO_2_^−^ flask^−1^ at ~33 h (Fig. 1a). Net production of N_2_O and NO was recorded (70–260 nmol flask^−1^) until nitrate and nitrite were depleted at ~55 h and ~80 h, respectively. Once the nitrogen oxyanions were depleted, NO and N_2_O concentrations decreased rapidly to zero, and the cumulated N_2_ production reached a stable plateau at 120 μmol flask^−1^ after ~80 h (Fig. 1c), which shows that all the provided nitrate was reduced to N_2_.

Methane production was detected at about 90 h, thus about 10 h after the cease in N_2_ production, while the CO_2_ production slowed down (Fig. 1d). At 180 h there was 141.2 nmol methane and 136 μmol CO_2_ flask^−1^, meaning that 65% of the quinoline was mineralized (initial amount of quinoline per flask was 23.26 µmol; equivalent to 209.3 µmol quinoline-C). According to the stoichiometry (equation 1) for denitrification-based mineralization, there should be 1.125 mol CO_2_ per mol N_2_-N produced by reduction of NO_3_^−^. Since all nitrate (initial amount 105 μmol flask^−1^) was recovered as N_2_-N, the denitrification should sustain a production of 118.1 μmol CO_2_, which is comparable to the level of cumulated CO_2_ production. It should be noted that there was an apparent decline in CO_2_ production from time 60 to 75 h, which is most probably due to a slight increase in pH by the fast reduction of nitrite in this period (Fig. 1). This could not be taken into account when calculating the amounts of CO_2_ per vial because pH was not monitored continuously. The results suggested that respiratory metabolism by denitrification largely accounted for the measured CO_2_ production, while fermentation did not significantly contribute to the CO_2_ production.

1 $${{\rm{C}}}_{9}{{\rm{H}}}_{7}{\rm{N}}+10{{\rm{O}}}_{2}\to 9{{\rm{CO}}}_{2}+{{\rm{NH}}}_{3}+2{{\rm{H}}}_{2}{\rm{O}}$$

### Profiling of the bacterial community by pyrosequencing

Samples were taken after 0 h, 25 h, 80 h, 144 h of incubation to study the dynamics of the microbial community composition, analyzed both at rDNA and rRNA level. The 454 pyrosequencing yielded 165,698 high-quality reads with an average of 6,904 reads per sample (±1532 SD) (Supplementary Fig. S1). In total, 489 species-level operational taxonomic units (OTUs) were delineated using 97% as a homology cut-off value. The richness was estimated by rarefaction and Chao 1, and diversity was estimated by Shannon diversity index and Simpson diversity (Supplementary Fig. S2). Bacterial diversity decreased over time, and much higher diversity was found in the amplicons of rDNA, comparing to rRNA sample.

**Figure 2:**
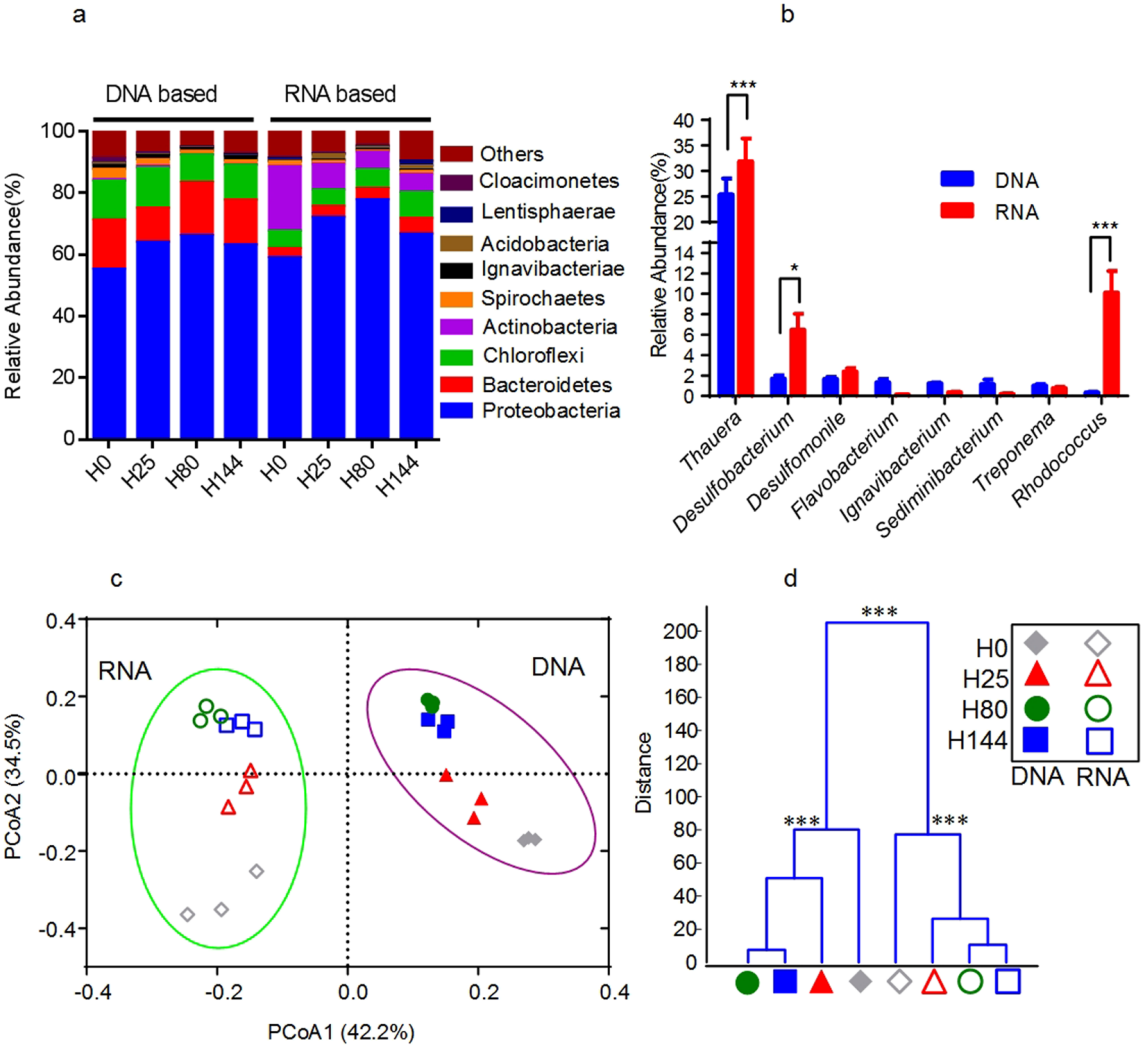
Over all structural changes of microbiota community during quinoline-degrading process based 16S rRNA gene V1-V3 454 pyrosequencing. Comparison of relative abundances of the major phylotypes (relative abundances above 1%) found in the DNA and RNA data. (**a**) At phylum level (**b**) at genera level. (**a**) The left part represents the phylotypes from the DNA, and the right part represents the phylotypes from the RNA-based data. The colors correspond to the major phylogenetic groups of the phylotypes. The different parts of the stacked bars represent the phylotypes identified. (**b**) The different stars above the columns mean significant differences as assessed by Tukey’s test (*P < 0.05, ***P < 0.001). (**c**) Bray-curtis PCoA of the quinoline-degrading microbiota at time H0, H25, H80 and H144 based on pyrosequencing OTU (97% identity) data. The values in parenthesis indicate the percentage of community variation explained by the axes. (**d**) Clustering of microbiota based on mahalanobis distances between different groups calculated with multivariate analysis of variance test, ***P < 0.001.

Most of the reads (93.5%) were assigned to 20 different phyla by RDP classifier, but only 49.3% of the reads could be assigned to known genera (data not shown). The five most dominant bacterial phyla included *Proteobacteria* (65.1%), *Bacteroidetes* (10.0%), *Chloroflexi* (9.0%), *Actinobacteria* (4.7%) and *Spirochaetes* (1.5%) (Fig. 2a). However, 31 OTUs contributed to 83.2% of all reads and constituted the predominant quinoline-degrading consortia members (see Supplementary Table S1). The genera that showed the highest metabolic activity, as seen from the rRNA analysis, were *Thauera* (31.8%), *Rhodococcus* (10.1%) and *Desulfobacterium* (6.5%) (Fig. 2b). *Thauera* species were enriched to be dominant populations with relative abundances of 38.7% in DNA library, and with supposedly high activity constituting 50.1% of rRNA sample at 80 h (Fig. 2a and Supplementary Table S2).

Principal coordinate analysis (PCoA) of the Bray-Curtis distance matrix based on OTUs was used to show the dynamics of the microbial community during quinoline-degrading process (Fig. 2c). The rDNA and rRNA-based differences were mainly observed along the first principal coordinate (PCoA1), which accounted for 42.2% of total variation. The second principal coordinate (PCoA2), accounting for 34.5% of the total variance, was associated with the time-related changes in the composition quinoline-degrading consortia. The PCoA2 clearly separated samples obtained at 0 h from those obtained at 25 h, 80 h and 144 h. Analysis of rDNA revealed a relatively stable composition and less temporal variation compared to the rRNA-derived communities. These findings were confirmed by clustering analysis based on distances calculated using multivariate analysis of variance (MANOVA) (Fig. 2d).

### Key species associated with quinoline-degradation

**Figure 3:**
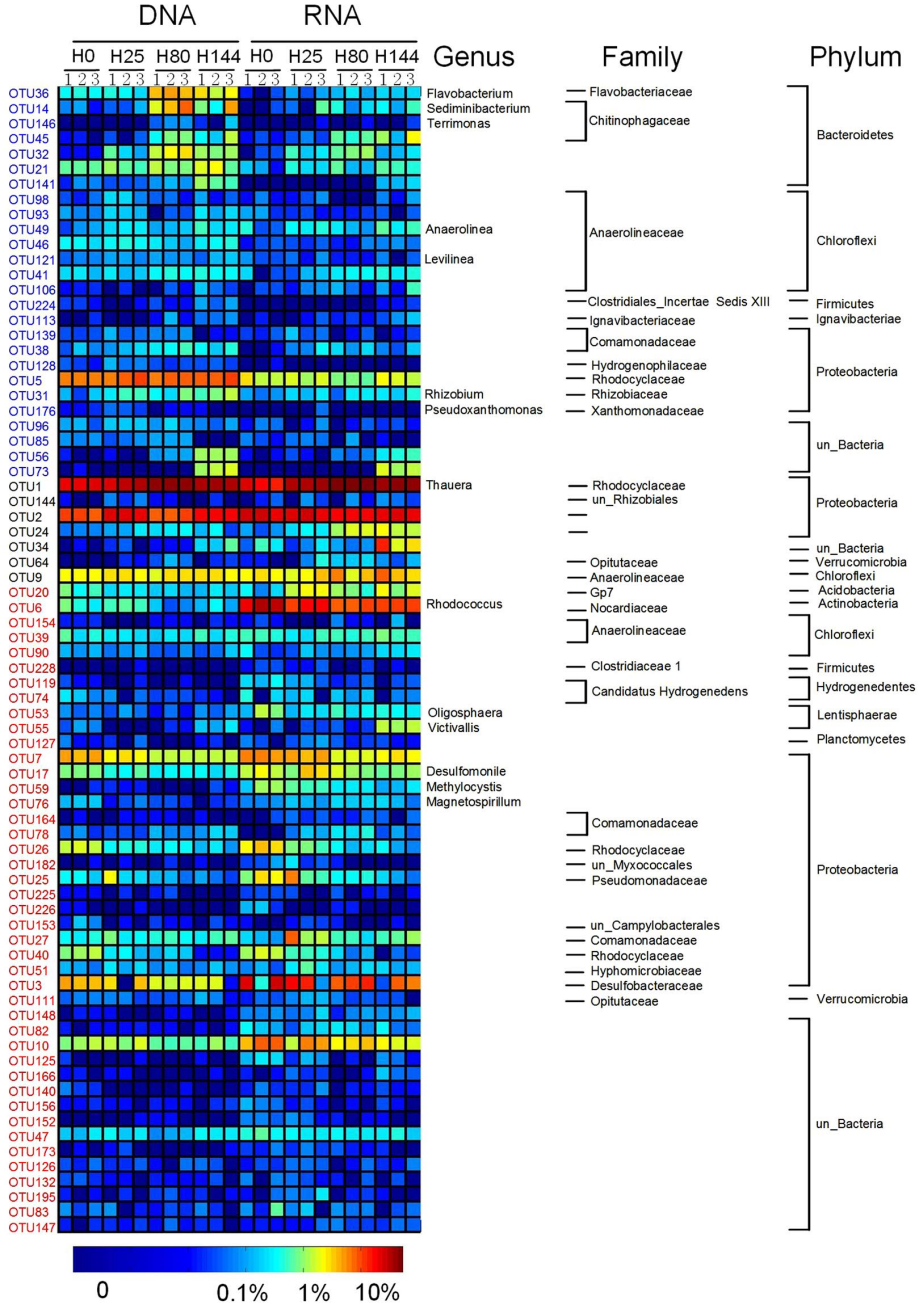
Key phylotypes (76 OTUs) associated with quinoline degradation detected by LEfSe. The color of the spot corresponds to the normalized and log-transformed relative abundance of the OTU. The taxonomy of the OTUs (genus, family and phylum) is depicted on the right.

During the quinoline degradation, the nitrogen electron acceptors were depleted sequentially, and finally entered into methanogenesis phase after ~80 h. Altogether 76 non-redundant OTUs, which apparently participated in quinoline degradation, were identified by LEfSe analysis, including 33 enriched OTUs at the DNA level and 50 active OTUs at the RNA level (Fig. 3, Supplementary Fig. S4 and Tables S2, S3).

At the DNA level, 17 OTUs were significantly enriched at 80 h when quinoline was almost removed, distributed among *Proteobacteria* (5 OTUs), *Bacteroidetes* (6 OTUs) and *Chloroflexi* (3 OTUs). Notably, only five OTUs were enriched at all sampling occasions, including OTU1 (*Thauera*), OTU31 (*Rhizobium*), OTU32 (*Bacteroidetes*), OTU49 (*Anaerolinea*) and OTU139 (*Acidovorax*). We also noticed that 7 OTUs were only enriched at 25 h, belonging to members of *Thiobacillus*, *Anaerolineaceae* and *Pseudoxanthomonas*; while 8 OTUs significantly increased at 144 h (methanogenic phase), including members of *Rhodocyclaceae*, *Anaerolineaceae* and *Ignavibacteriaceae*. (Supplementary Fig. S4, Table S2).

At the RNA level, a total of 50 OTUs were identified as active phylotypes, which shared 7 enriched OTUs in the DNA analysis, including OTU1 (*Thauera*), OTU2 (*Syntrophobacteraceae*), OTU9 (*Anaerolineaceae*), OTU24 (*Burkholderiales*), OTU34 (unclassified bacteria), OTU64 (*Opitutaceae*) and OTU144 (*Rhizobiales*). At 144 h, OTU55 (*Victivallis*), OTU9 (*Anaerolineaceae*) and OTU90 (unclassified *Chloroflexi*) showed higher activity. These active phylotypes might live from fermentation of compounds resulting from quinoline degradation.

### Co-occurrence pattern of key phylotypes in the consortia

**Figure 4:**
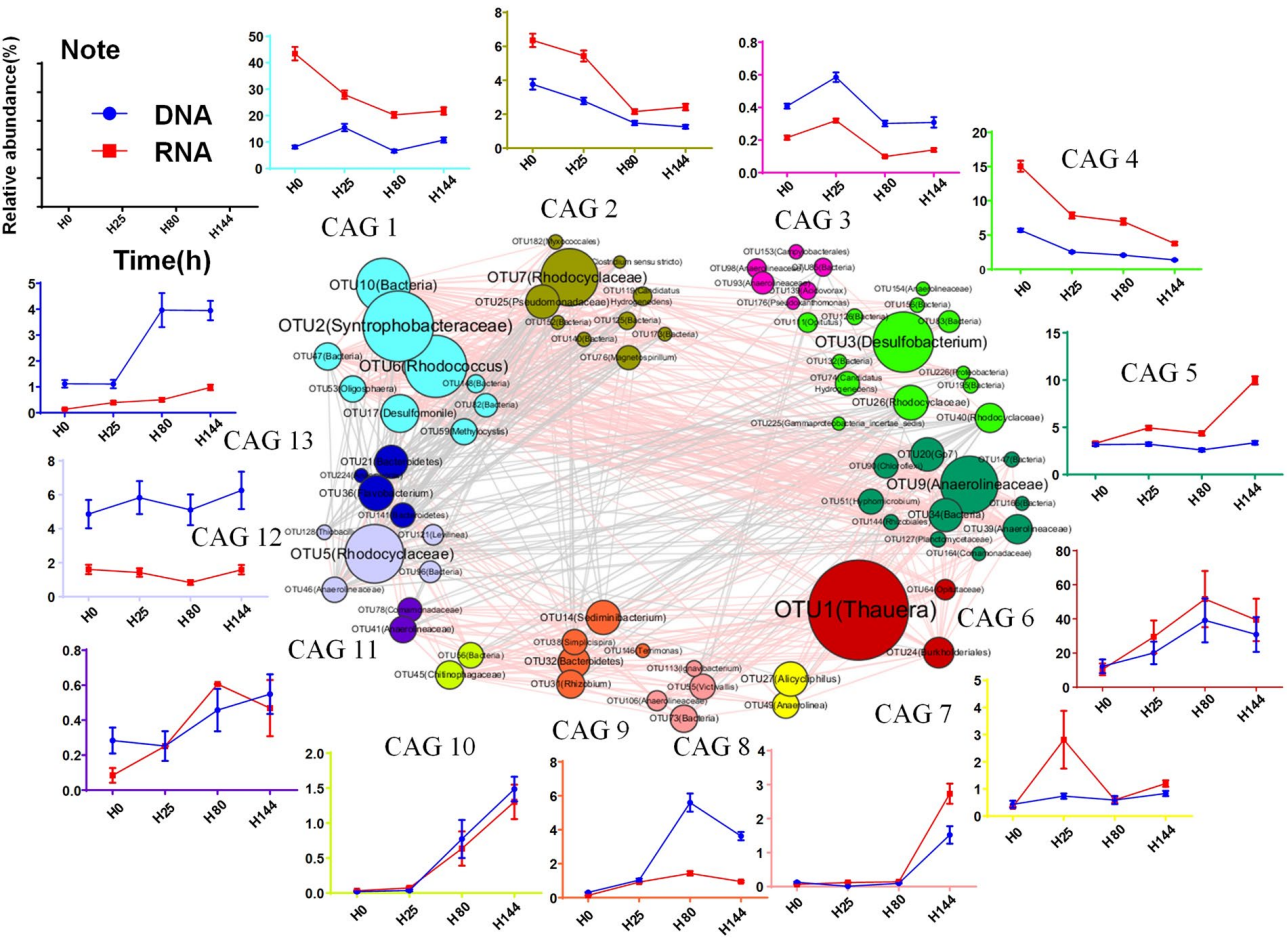
Temporal dynamics of the microbiome during the incubation process. OTU-level network diagram of 76 key OTUs responding to the quinoline degradaion process. Node size indicates the mean abundance of each OTU. Lines between nodes represent correlations between the nodes they connect, with the colour saturation indicating correlation magnitude: pink represents positive correlation, grey represents negative correlation. Only lines corresponding to correlations with a magnitude greater than 0.4 are drawn. The OTUs are grouped into 13 CAGs by permutational multivariate analysis of variance (PERMANOVA) when P < 0.05. The plots show the abundance of each CAG on H0, H25, H80 and H44 in DNA and RNA level. Data in plots represent the total abundance of all OTUs in each CAG from each sample, which were then visualized by mean ± s.e.m.

Co-occurrence networks produced from sequencing data are frequently used to identify interactions between community members. In present work, cooperation and competition between OTUs were analyzed by generating a correlation network. The 76 key OTUs were clustered into 13 co-abundance groups (CAGs) based on SparCC correlation coefficients (Fig. 4). Plotting the corresponding CAG abundances over time demonstrated a strongly shared dynamic relationship across time, and across nearly all individuals, for many specific CAGs involved in the quinoline degradation process. Presence of quinoline enriched CAG6, 8, 9, 10, 11, 12 and 13 and decreased CAG2 and CAG4. The amount of CAG1, 3, 5 and 7 was nearly constant across time. CAG6 was a core CAG, which showed fast growth. It only included three members, OTU1 (*Thauera*), OTU24 (*Burkholderiales*) and OTU64 (*Opitutaceae*). An earlier increase was observed in CAG1 and CAG3 at the rDNA level (25 h), after which the abundance of these group decreased, suggesting that they were involved in the start of the denitrifying quinoline degradation. CAG1, the second largest group contained 10 OTUs, mainly *Syntrophobacteraceae* (OTU2) and *Rhodococcus* (OTU6), showed higher activity in the rRNA level. During the methanogenesis phase (144 h), the abundance of CAG5, CAG8 and CAG12 increased. The abundance of CAG2 and CAG4 continually decreased over time, but showed remarkable activity.

### Correlation between 76 key OTUs responding to physiological characteristics

**Figure 5:**
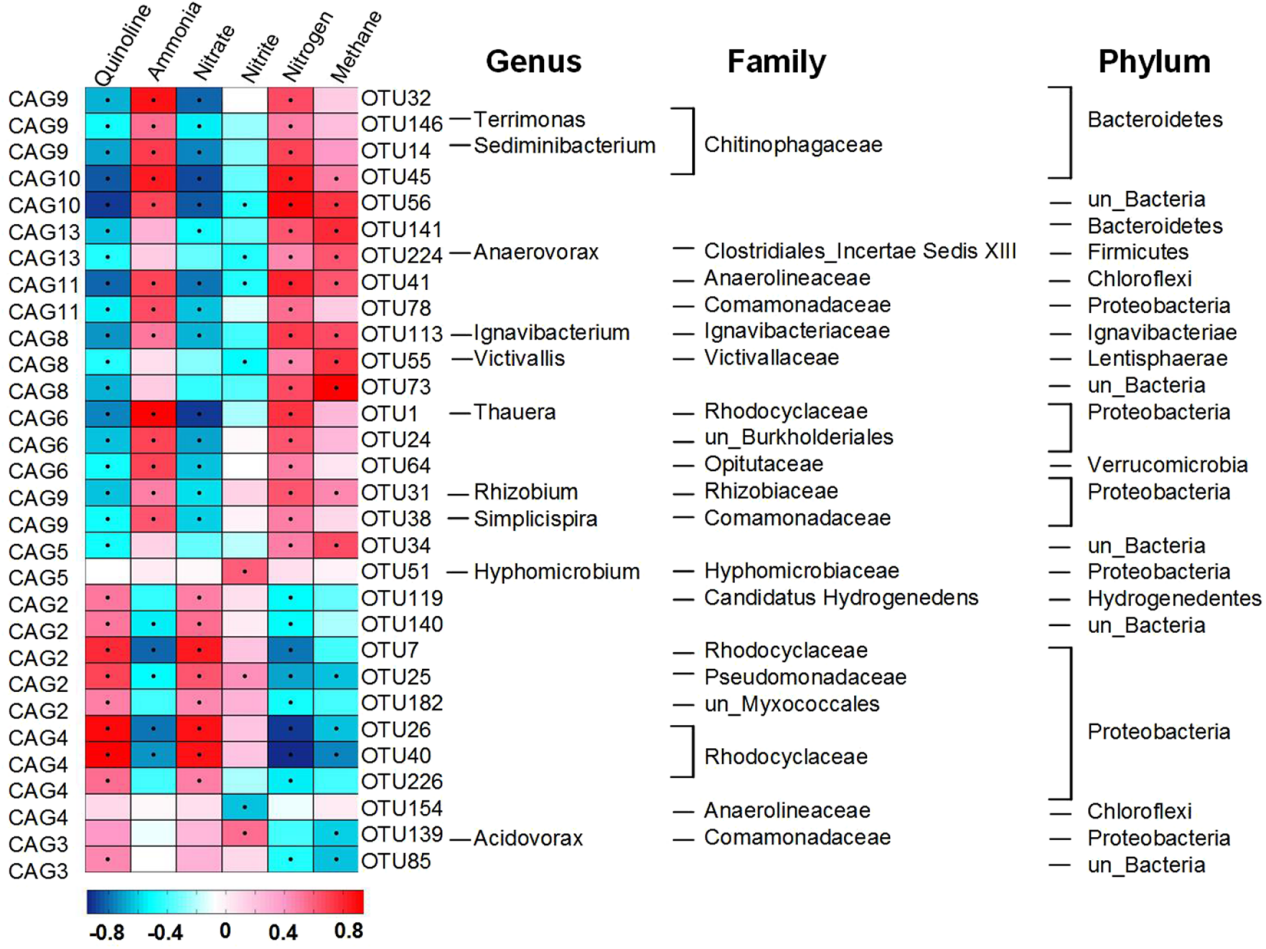
Thirty OTUs that were significantly correlated with physiological parameters during quinoline degradation. Rows correspond to OTUs with the IDs shown on the right, the co-abundance group (CAG) IDs was on the left. The columns correspond to physiological parameters related to the concentration of quinoline (mg/L), ammonia (mg/L), nitrate (mg/L) and ammonia (mg/L), the accumulated nitrogen (μmol) and methane (nmol). Red and blue colors denote positive and negative association, respectively. The intensity of the colors represents the degree of association between the OTU abundances and physiological parameters as assessed by the Spearman’s correlations. The black dots in the blue/red cells indicate that the associations were significant (false discovery ratio < 0.05). The taxonomy of the OTUs is shown as phylum, family and genus level.

In this study, we aimed to link specific organisms with their potential roles in anaerobic quinoline biodegradation. Spearman’s correlation analysis was performed between the 76 OTUs and physiological parameters. In total, 30 OTUs were significantly correlated with at least one physiological parameter (Fig. 5 and Supplementary Table S4). Nineteen of these key OTUs were positively correlated with quinoline removal, nitrate removal and nitrogen accumulation, which represents important denitrification phenotypes such as OTU1 (*Thauera*), which showed a strong positive correlation (R > 0.7, p < 0.01) with quinoline removing efficiency. Thirteen key OTUs were correlated with increasing amounts of ammonia, and ten key OTUs were positively correlated with methanogenesis, including OTU73 (unclassified bacteria), OTU141 (*Bacteroidetes*), OTU41 (*Anaerolineaceae*) and OTU55 (*Victivallis*). They are presumed to be fermentative bacteria because no exogenous electron acceptor existed at this stage. Especially in CAG8, three OTUs showed a high correlation with methane production(R > 0.6, P < 0.01).

## Discussion

In this study, we obtained a detailed picture of quinoline degradation under denitrifying conditions in a batch experiment using an inoculum from a quinoline degrading, anaerobic bioreactor. Earlier studies showed that quinoline biodegradation proceeded in the presence of nitrate, but came to a halt when the nitrate was depleted^10^, while Burland *et al*. found that benzene oxidation was more tightly coupled to incomplete reduction of nitrate to nitrite rather than its complete reduction to N_2_^17^. In our experiments, we noted that all of the NO_3_^−^ was reduced to N_2_ before the start of methanogenesis, suggesting that the diverse denitrifying bacteria in the consortium performed an effective nitrite reduction and complete denitrification.

Most of the quinoline (83.5%) had disappeared during the denitrification stage. If completely mineralized, this should theoretically produce 178 µmol CO_2_, which is more than that measured at the time of nitrogen oxyanion depletion. However, the cumulated CO_2_ production may have been underestimated due to the pH increase driven by denitrification. The accumulated CO_2_ showed that respiratory metabolism by denitrification was the main degradation process, and the recovery of quinoline C at this stage suggested nearly complete mineralization. Until now, in the transformation study of quinoline and its derivatives under denitrifying, only two intermediate metabolites were identified (1H-2-oxoquinoline and 3,4-dihydro-1H-2-oxoquinoline)^10,11^. In this study, we also failed to detect any intermediate aromatic metabolites. These results suggested that the denitrifying bacteria in the consortium cooperated very efficiently in degrading the quinoline after a long-term adaptation in our bioreactor.

In the present study, *Thauera* (OTU1, the family *Rhodocyclaceae*), was overwhelmingly enriched during the process of 80 h incubation. Bacteria belonging to the genus *Thauera* have been reported as important quinoline-degraders under nitrate-reducing conditions^14^. The closest described representative of OTU1 was *Thauera sp*. MZ1T (100% rRNA gene sequence identity), which was isolated from activated sludge samples^18^. Most members of *Thaurea* are known as nitrate-reducing bacteria, and several species have been characterized for their ability to anaerobically degrade various aromatic compounds^19,20^. In our experiment, it is notable that the *Thauera* exhibited a much larger and immediate increase compared to all other community members at 25 h. This suggested that OTU1 (*Thauera*) was most likely involved in the initial step of quinoline degradation.

Syntrophy plays a key role in many anaerobic ecosystems, even in systems where respiratory metabolism is sustained by reduction of electron acceptors such as sulphate or nitrogen oxides^21,22^. In this study, phylogenetic analysis of the community showed that, aside from the genus *Thauera*, members of the families *Rhodocyclaceae*, *Syntrophobacteraceae*, *Desulfobulbaceae* and *Anaerolineaceae* also dominated and increased their abundance during the quinoline degradation. These lineages were also reported as the most frequently encountered bacterial taxa in benzene/toluene degrading cultures^22,23^. A summary of the key bacterial taxa associated with syntrophic aromatic hydrocarbon-degrading consortia (mainly benzene) was provided by Kleinsteuber *et al*.^22^. They identified bacteria belonging to the sulfate-reducing delta- *Proteobacteria* group (*Syntrophobacteraceae* and *Desulfobulbaceae*) as acetate-consuming or hydrogenotrophic organisms that utilize metabolites released during benzene fermentation, while *Chloroflexi*, *Anaerolineae*, and *Bacteroidetes* were considered to be secondary degraders that degrade dead biomass or hydrocarbon intermediates, in addition to scavenging acetate and H_2_.

In time course studies, analysis of the co-occurrence patterns of OTUs and correlations of these patterns with specific environmental factors could give an insight into community structure-function relationships^24,25^. In our co-abundance network analysis, the OTUs having similar eco-physiological characteristics were clustered together^25,26^. Altogether, 13 CAGs were identified in the batch culture of this study. Of these, CAG6 had the fewest members and was dominated by OTU1 (*Thauera*), which seems to represent a core bacterium with a possibly irreplaceable role in the consortium.

There were only two other, low abundance teammates in CAG6, OTU24 (*Burkholderiales*, 0.18%) and OTU64 (*Opitutaceae*, 0.03%). Along with the *Thauera* group, OTU24 and OTU64 might also be involved in the primary attack of the quinoline molecule, but their low abundance suggested that they were not as competitive. *Burkholderia* species that are capable of degrading polyaromatic hydrocarbons have been frequently isolated^27^.

The 73 key phylotypes identified in the other 12 CAGs showed different dynamic patterns across time and were diverse in classification, suggesting that these species played different roles in the degradation process, probably by using different intermediates. Some CAGs only responded during particular quinoline degradation phases. When all the electron acceptors were exhausted, some significant active group were observed at 144 h, mainly including *Anaerolineaceae* (OTU9), *Rhodocyclaceae* (OTU5) and other low abundance OTUs, which could be identified as fermentative bacteria to remove remain carbon substrates at methanogenesis phase.

Another observation was that, unlike the significant enrichment of *Thauera* in CAG6, the relative transcript abundances of CAG1, 2 and 4, which were dominated by *Syntrophobacteraceae* (OTU2), *Rhodococcus* (OTU6), *Rhodocyclaceae* (OTU7) and *Desulfobacterium* (OTU3), decreased during quinoline degradation despite their apparent higher activity as judged from their abundance in the RNA data. In contrast to continuous-flow reactors, where intermediate metabolites are supplied in a sustained way to support the activity of fermentative bacteria, the amounts of intermediate metabolites will eventually decrease due to substrate exhaustion. These intermediate metabolites were most likely used up by members of CAG1, 2 and 4, which would explain the relatively high but decreasing abundance of these groups. Members of the *Syntrophobacteraceae* are mainly found in freshwater, sewage sludge or marine habitats, and have been described as obligate anaerobes that can degrade long-chain fatty acids and propionate^28,29^. They were also observed *in situ* in a benzene-degrading microcosm where they utilized metabolites released from benzene degradation^22^. Members of *Desulfobacterium* are known to be metabolically versatile^30^, and the *Desulfobacterium indolicum* (DSM 3383) was shown to degrade quinoline under sulfate-reducing conditions. *Desulfobacterium*-like species (OR-M2) might play an important role in benzene-degrading methanogenesis process^31^. The genus *Rhodococcus* was regarded as one of the most promising organisms suitable for the biodegradation of recalcitrant compounds^32^. We isolated several strains of *Rhodococcus* from the studied bioreactor, but none of them could use quinoline as sole carbon source under denitrifying conditions (X Zhang, unpublished data). It is therefore likely that these bacteria utilize intermediate metabolites released during the incubation.

Meanwhile, it was worth noting that most of the detected *Chloroflexi* population (8.9%, 53 OTUs) was assigned to the class *Anaerolineae* (46 OTUs) and only 12 OTUs were identified to genus of *Anaerolinea*, *Bellilinea* and *Levilinea*, which were all low abundance species (<0.5%). The OTU8 and OTU9 (genus unclassified) dominated the family by higher relative abundance (4.8% and 2.0% at rDNA level, respectively). All the currently characterized species of the class *Anaerolineae* are anaerobic bacteria that decompose carbohydrates via fermentation^33^. Numerous studies have demonstrated the occurrence of members of *Chloroflexi* (mainly *Anaerolineaceae*) in many oil and hydrocarbon environments^34^, accounting for up to nearly half of the prokaryotic community in several reactors^33^. These bacteria were associated with anaerobic n-alkane degradation and benzene/toluene degradation. Moreover, it was inferred that members of *Anaerolineae* were involved in carbohydrate fermentation in these anaerobic systems and that they then were providing organic acids such as acetate to other microorganisms. In our quinoline-degrading consortia, members of *Anaerolineae* were distributed over 8 CAGs and showed different dynamic patterns. Therefore, it is likely that as secondary fermenters they contributed to the removal of multiple intermediate metabolites.

In the batch culture, most of the quinoline was degraded during the denitrification stage, where nitrate was reduced to dinitrogen gas. Based on the potential roles of organisms in the batch culture consortium discussed above, we propose a model for the involvement of bacteria in the quinoline degradation (Supplementary Fig. S5). *Thauera* as core bacteria appeared to be responsible for the initial attack on quinoline; members of *Desulfobacterium*, *Rhodococcus*, *Rhodocyclaceae* and *Syntrophobacteraceae*-related phylotypes utilized metabolites released from the early phase of quinoline degradation; and *Anaerolineae* populations, as secondary fermenters, contributed to the degradation of the later intermediate compounds. The information provided in this study identifies the bacterial groups responsible for quinoline degradation under denitrifying conditions and improves the understanding of the kinetics of this process.

## Materials and Methods

### Operation of the bioreactor

An anaerobic reactor, inoculated with seeding sludge collected from an anoxic tank of a coking wastewater treatment plant, was operated with quinoline as sole electron donor and nitrate as electron acceptor. The 2 L reactor, was fed continuously with synthetic wastewater composed of quinoline (100 mg/L), NaNO_3_ (240 mg/L) and K_2_HPO_4_ (140 mg/L), was incubated in a temperature-controlled room at around 25 °C. The hydraulic retention time was 24 h. During the incubation, microbial consortia developed as biofilms that floated in the fluid or were attached to hollow plastic carriers.

### Preparation of inoculum and experimental setup

Biofilm samples from the nitrate-reduction quinoline-degradation bioreactor were used as inoculum for batch experiments. Briefly, 2 g of biofilm sample was scratched off from the surfaces of plastic carriers with a sterile tweezer. The sample were suspended in sterile centrifuge tubes containing glass beads and 40 mL liquid from the bioreactor. The suspensions were used as inocula in the subsequent experiments after being homogenized by vortexing.

The batch experiments were performed in 120 ml serum bottles containing 30 ml medium [100 mg quinoline, 297.6 mg NaNO_3_ and 140 mg K_2_HPO_4_ l^−1^]. After inoculation with 0.5 ml seed culture, the bottles were immediately sealed with air-tight butyl-rubber septa and aluminum crimp caps and made anoxic by repeated evacuation and filling with helium (He). Altogether 21 replicate bottles were prepared and all incubations were in the dark at 28 °C. Three of these bottles were kept intact to monitor gas kinetics of the N_2_, N_2_O, NO, CO_2_ and CH_4_ during 400 h. Three uninoculated control bottles were also included. Three bottles were used for chemical monitoring, from which 2 ml of suspension were sampled with syringes (needle through septa) periodically (0 h, 12 h, 25 h, 33 h, 45 h, 55 h, 80 h, 120 h, 144 h). All samples were centrifuged to precipitate microbial cells. The supernatant was used to determine the concentration of quinoline, nitrate, nitrite, ammonia and pH. The remaining 12 bottles were incubated in a shaker and sacrificed for nucleic acid extraction after 0, 25, 80 and 144 h.

### Gas measurements

A robotized incubation system was used to measure gas kinetics^35,36^, which allowed direct quantification of gas production (NO, N_2_O, N_2_,CO_2_ and CH_4_) during the quinoline degradation process. In brief, the robotized system comprises an incubation system and a gas collection and analysis system. It allows repeated sampling of headspace gas by piercing the butyl rubber septa of the reaction serum flask, and pumping sample gas by a peristaltic pump through the injection loop of an Agilent 7890 A gas chromatography and further to a NOx analyser (GE NOA 280i, GE Company, USA) for measuring gas. Sampled gas (2 mL per sampling) was replaced by injection of He, and this dilution was taken into account when calculating the production of N_2_, CO_2_ and CH_4_. Headspace gas samples were collected every 2 h by the automated sampling system described above. Dilution and leakage were taken into account when estimating gas transformation rates for each time increment between two samplings.

### Chemical analyses

Samples were centrifuged at 10,000 g for 10 min to remove the cells. The quinoline concentration was measured using high-performance liquid chromatography according to a previously described method^16^. Nitrate was measured by rapid spectrophotometric determination using phenol^11^. Nitrite was measured using the N-(1-naphthalene)-diaminoethane photometry method and ammonia was determined by photometry with Nessler’s reagent^37^.

### Biodegradation kinetic model analysis

Numerous models have been used to describe the biodegradation kinetics of organic pollutants, and the first-order kinetic have been used frequently to describe biodegradation progress at low substrate concentrations^38,39^. The quinoline biodegradation is assumed to fit to the first-order kinetic reaction, which has the following equation:

$${{\rm{lnQ}}}_{{\rm{t}}}=c-{\rm{kt}}$$

where Q_t_ designates the concentration of quinoline at time t; k is the first-order kinetic constant; t is time and c is a constant. Only when Q_0_ ≪ Ks (Ks is the half-saturation rate coefficient), the equation was tenable.

### Nucleic acid extraction, reverse transcription and 454 pyrosequencing

After sacrificing bottles at successive time points of the experiments, 20 ml of the cultures in each flask was collected for RNA extraction and the remaining 10 ml of the cultures was used for DNA extraction. The cultures were centrifuged at 12, 000 g at 4 °C for 2 min (Heraeus Instruments, Germany). The pellets for RNA extraction were treated with RNAlater^®^ RNA Stabilization Solution from Ambion. Genomic DNA was extracted using the method described by Godon *et al*.^40^. Total RNA extraction was performed with the RNA PowerSoil^®^ Total RNA Isolation Kit (MoBio) following the manufacturer’s instructions. Contaminating DNA was removed using the DNase I (Invitrogen Life Technologies, Carlsbad, CA, USA) digestion according to the manufacturer’s instructions, and DNA contamination was tested on all samples by PCR with primers targeting the 16S rRNA gene. The nucleic acid extracts were checked for quality by standard agarose gel electrophoresis and ethidium-bromide staining, and concentrations were determined by a spectrophotometer (BioDrop Technologies). For each sample, 100 ng total RNA was reverse transcribed to cDNA using SuperScript^TM^ III First-Strand synthesis system (Invitrogen) following the manufacturer’s instructions with random hexamers.

The DNA and cDNA from each sample was used as the template to amplify the V1-V3 region of 16S rRNA genes. We multiplex-sequenced the resulting amplicons with a barcoded primer strategy on a 454 Genome Sequencer FLX-Titanium platform using the manufacturer suggested protocol. The forward primer PF002, 5′-CGTATCGCCTCCCTCGCGCCATCAG-ACGCTCGACA-AGAGTTTGATYMTGGCTCAG-3′; and reverse primer PRxxx, 5′-CTATGCGCCTTGCCAGCCCGCTCAG-NNNNNNNNNN-ATTACCGCGGCTGCTGG-3′. The bar-coded 10-base ID tag in the reverse primer, which was 10 ‘N’ in the middle of reverse primer, was used to distinguish PCR products from different samples by different ID sequences. PCR amplification, pyrosequencing of the PCR amplicons and quality control of raw data were performed as described previously^41^. Briefly, we performed the following steps to select the high quality reads for bioinformatics analysis: (a) 3′ ends were trimmed when the average Phred quality score of a sliding window of 50 nt dropped below 25, (b) forward primer was detected, (c) after trimming the primer and barcode bases, those sequences with a variable region more than 300 nt and less than 700 nt in length and with no more than two undetermined bases were preserved.

### Bioinformatics analysis of pyrosequencing data

All high-quality sequences were extracted, aligned in Greengenes to remove sequences with less than 75% identity with bacteria. OTU classification was performed using UPARSE^42^, briefly, high-quality sequences were dereplicated into unique sequences, and singletons were discarded, representative OTU sequences were next picked. Further chimera detection was performed using UCHIME^43^ against the RDP classifier training database. The OTU table was finalized by mapping quality-filtered reads to the remaining OTUs with the Usearch global alignment algorithm at a 97% cutoff. The most abundant sequence of each cluster was subjected to online RDP classifier (Version 2.10) for taxonomical assignment with a bootstrap cutoff of 50% (http://rdp.cme.msu.edu/classifier/classifier.jsp). The representative sequences, together with the abundance data (normalized for each sample), were used for further analysis. The alpha and beta diversities were performed using QIIME (version 1.8)^44^. The Shannon-Wiener index, Simpson’s diversity index, and Chao1 and rarefaction estimates were calculated, and principal coordinate analysis (PCoA) was used to visualize the Bray-Curtis dissimilarity matrices based on the 97% OTU level across different time points. Sequence data are deposited in NCBI’s Sequence Read Archive (SRP068489). The statistical significance of the separation among groups was assessed by MANOVA using the PCoA scores in MATLAB 2010b (The MathWorks, Inc., USA). Microbial communities were analyzed in two different ways: (1) changes in microbial community composition over time; and (2) differences between microbial community profiles given by rDNA and rRNA samples.

LEfSe^45^, an online algorithm for high-dimensional biomarker discovery was used to identify specific bacterial phylotypes that contributed to the quinoline degradation (http://huttenhower.sph.harvard.edu/galaxy). The differential features were identified on the OTU level. Briefly, first on DNA level, time points were used as the class of subjects (0 h via 25 h, 80 h, 144 h, respectively), which showed highest level of abundances in sample 25 h, 80 h and 144 h were selected. Secondly, active bacterial phylotypes were selected through pairwise comparisons were performed at four time point (0 h, 25 h, 80 h and 144 h) by DNA via RNA sample. We examined co-occurrence patterns using network analysis. For the input matrices we only considered OTUs that occurred in at least 20% of the samples and abundance >0.01%. Correlation between OTUs was calculated with the Sparse Correlations for Compositional data algorithm (SparCC) with a bootstrap procedure repeated 100 times^46^. Only 76 OTUs identified by LEfSe were represented in the network using Cytoscape (http://www.cytoscape.org) with R values less than 0.4 or larger than −0.4. The Ward clustering algorithm and PERMANOVA (9999 permutations, P < 0.05) based on SparCC correlation coefficients were used to cluster the 76 key OTUs into 13 co-abundance groups (CAGs) using the R program. Correlations between key 76 OTUs and individual physiological parameters, such as the concentration of quinoline, methane, ammonia, nitrate and nitrogen, were identified using Spearman’s correlation (MATLAB 2010b). False discovery rate control was used to account for multiple comparisons when evaluating correlations between OTUs and physiological parameters in Matlab software, and correlations were deemed significant at false discovery rate < 0.05.

## Electronic supplementary material

Supplementary information
