# Supplementary material for: Time-resolved analysis of a denitrifying bacterial community revealed a core microbiome responsible for the anaerobic degradation of quinoline
Source: Sci Rep. 2017 Nov 7;7:14778. doi: 10.1038/s41598-017-15122-0 (PMC5677008; doi:10.1038/s41598-017-15122-0)
Supplement: Supplementary file 1 — Supplementary information [file 41598_2017_15122_MOESM1_ESM.doc]

**Supplementary Information**

**Time-resolved analysis of a denitrifying bacterial community revealed a core microbiome responsible for the anaerobic degradation of quinoline**

Yun Wang^1,2^, Hao Tian^1^, Fei Huang^1^, Wenmin Long^1^, Qianpeng Zhang^1^, Jing Wang^1^, Ying Zhu^1^, Xiaogang Wu^1^, Guanzhou Chen^1^, Liping Zhao^1^, Lars R**.** Bakken^3^, Åsa Frostegård^4^, Xiaojun Zhang^1^*

^1^ State Key Laboratory of Microbial Metabolism and School of Life Sciences and Biotechnology, Shanghai Jiao Tong University, Shanghai 200240, PR China

^2^ Key Laboratory of Biogeography and Bioresource in Arid Land, Xinjiang Institute of Ecology and Geography, Chinese Academy of Sciences, Urumqi 830011, PR China

^3^ Department of Environmental Science, Norwegian University of Life Sciences, Ås N-1432, Norway.

^4^ Department of Chemistry, Biotechnology and Food Science, Norwegian University of Life Sciences, Ås N-1432, Norway.

* Corresponding author: Dr. Xiaojun Zhang

State Key Laboratory of Microbial Metabolism, School of Life Sciences & Biotechnology, Shanghai Jiao Tong University, Shanghai 20040, PR China.

Tel: +86 21-34204878; fax: +86 21-34204878; e-mail: xjzhang68@sjtu.edu.cn

**Supplementary Figure S1**

**Evaluation of the sequencing depth in each sample.** (a) Rarefaction curves of the samples. (b) Shannon diversity index curves of the samples. DNA samples is represented by a red line, while RNA samples is represented by a green line.


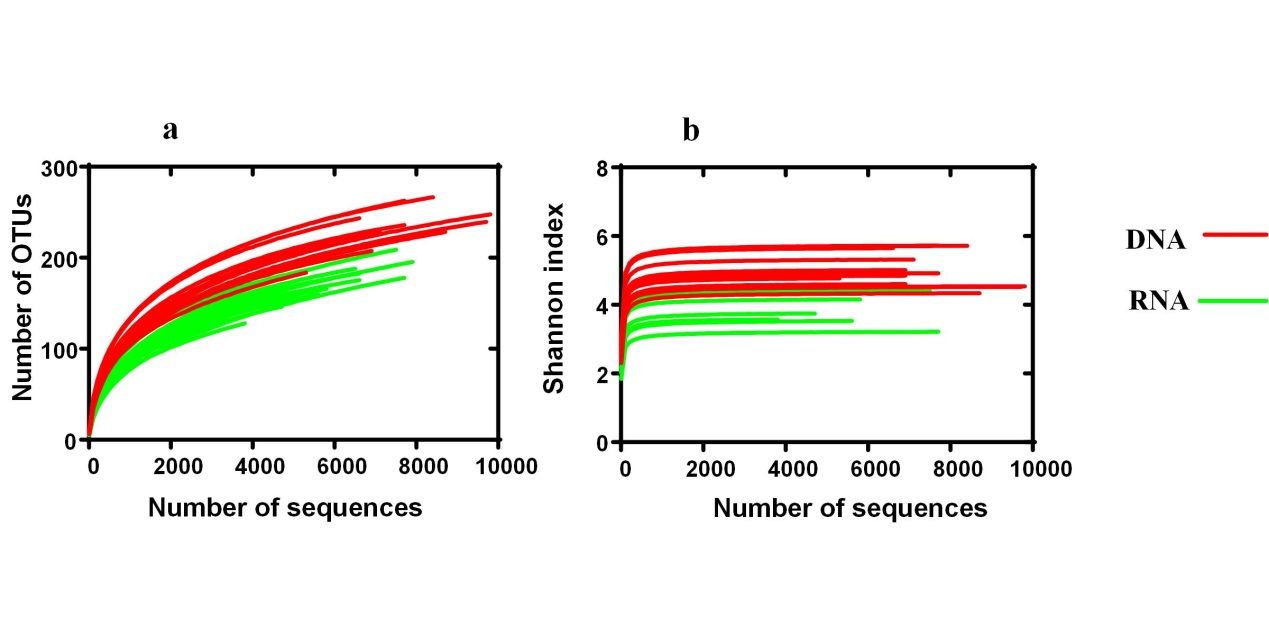


**Supplementary Figure S2**

**Richness and diversity of the quinoline-degrading microbiota.** (A)Shannon-Wiener index. (B) Simpson's index. (C) OTU estimates via rarefaction analysis. (D) Chao1 richness estimates. Calculations were performed after rarefying an equal number of sequence reads for all samples. Values are expressed as means±standard error.

**Supplementary Figure S3**

**Comparison of relative abundances of the major phylotypes (relative abundances above 1 %) found in quinoline degradation communities between DNA and RNA samples.** (a) at phylum level (b) at family level. Mean significant differences as assessed by Tukey’s test (*** P< 0.001).

**Supplementary Figure S4**

**Venn diagram showed key phylotypes identified by LEfSe.** (A) 33 significantly enriched OTUs identified using H0 sample as a baseline. (B) 50 significant active OTUs identified by comparing the DNA and RNA sample at H0, H25, H80 and H144.


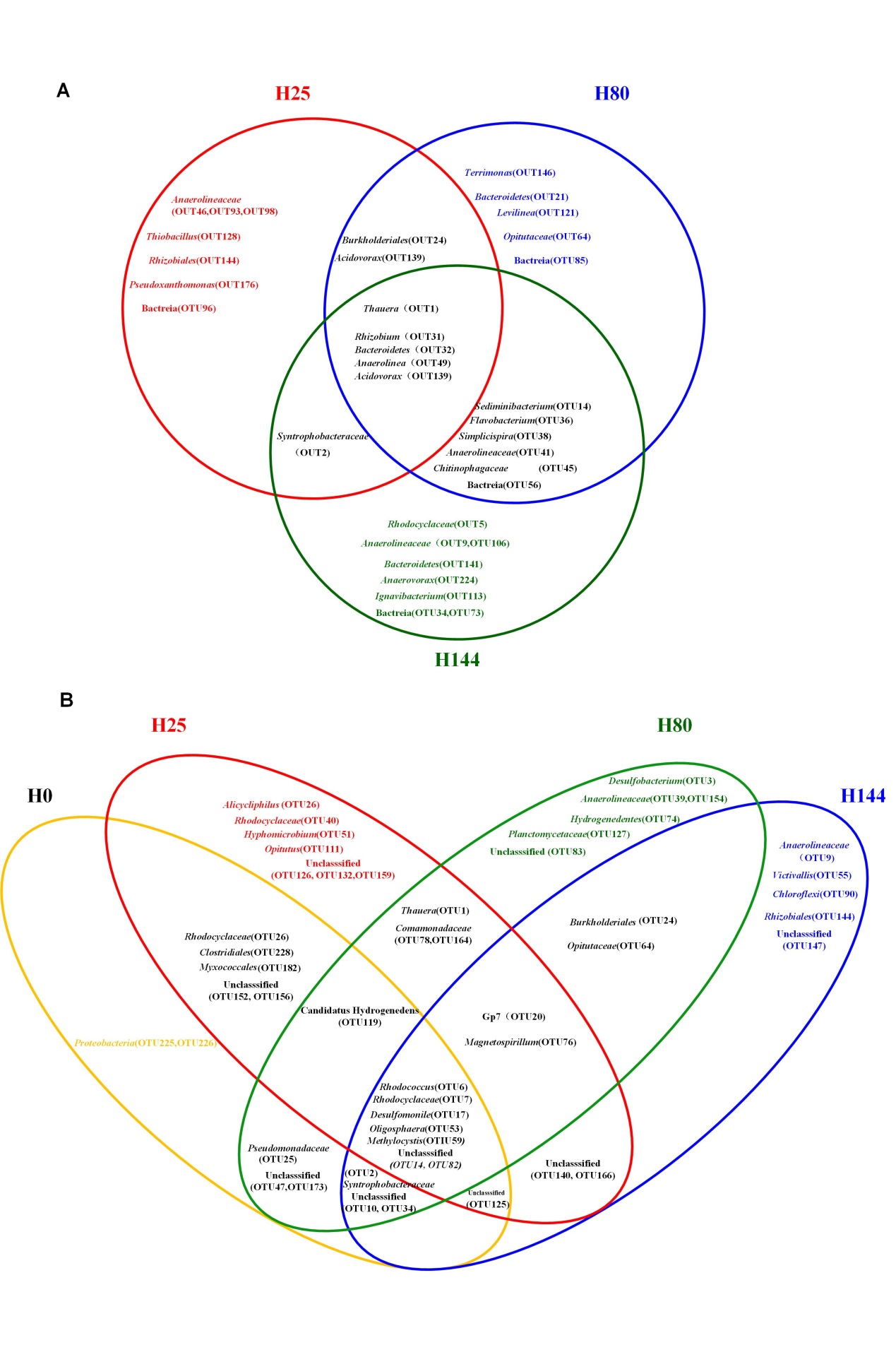


**Supplementary Figure S5 A hypothetic model of quinoline degradation**

**
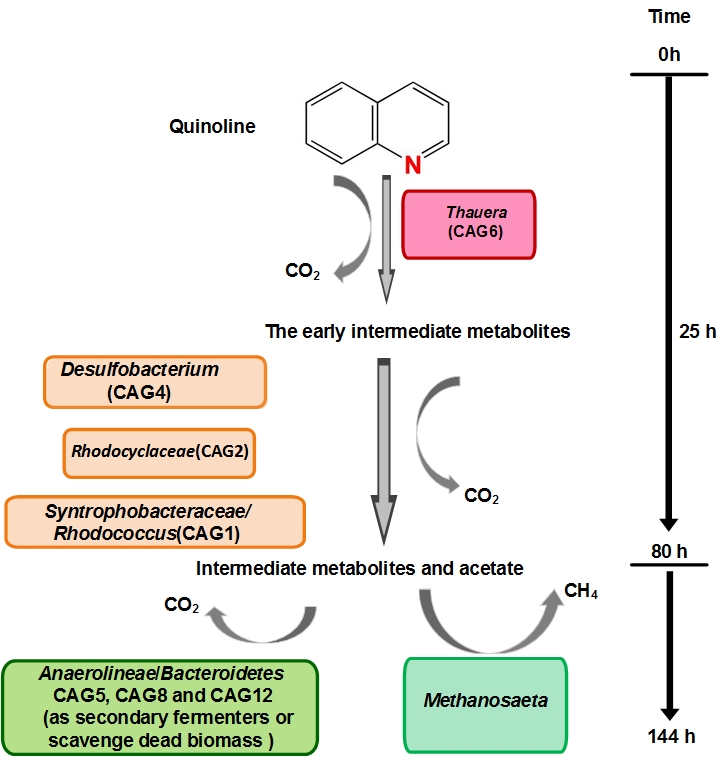
**

**Supplementary Table S1.**

**Summary of the relative abundances of OTUs** **in the quinoline-degrading nitrate-reducing cultures.** Only the most abundant 31 OTUs are displayed which covered more than 83.2% of raw sequences.

| **OTU ID** | **Taxonomical assignments** | | | | | **Relative abundance (%) （DNA,SD/RNA,SD)** | | | |
| --- | --- | --- | --- | --- | --- | --- | --- | --- | --- |
|  | **Phylum** | **Class** | **Order** | **Family** | **Genus** | **H0** | **H25** | **H80** | **H144** |
| OTU1 | *Proteobacteria* | *Betaproteobacteria* | *Rhodocyclales* | *Rhodocyclaceae* | *Thauera* | (12.17,0.87)/(10.36,4.07) | (19.89,4.66)/(29.07,3.56) | (38.78,2.67)/(50.10,3.74) | (30.68,2.50)/(37.92,5.76) |
| OTU4 | *Proteobacteria* | *Betaproteobacteria* | *Rhodocyclales* | *Rhodocyclaceae* |  | (8.94,0.29)/(2.56,2.24) | (8.47,1.55)/(2.75,0.40) | (4.24,0.21)/(1.10,0.09) | (5.83,0.20)/(1.56,0.31) |
| OTU5 | *Proteobacteria* | *Betaproteobacteria* | *Rhodocyclales* | *Rhodocyclaceae* |  | (4.32,0.08)/(1.45,0.51) | (5.03,0.93)/(1.29,0.29) | (4.64,0.39)/(0.74,0.03) | (5.65,1.05)/(1.43,0.26) |
| OTU7 | *Proteobacteria* | *Betaproteobacteria* | *Rhodocyclales* | *Rhodocyclaceae* |  | (3.20,0.25)/(3.88,0.21) | (1.87,0.19)/(3.02,0.16) | (1.29,0.03)/(1.43,0.03) | (1.15,0.04)/(1.98,0.12) |
| OTU19 | *Proteobacteria* | *Betaproteobacteria* | *Rhodocyclales* | *Rhodocyclaceae* |  | (2.27,0.28)/(1.31,0.27) | (1.66,0.07)/(1.47,0.37) | (1.12,0.23)/(0.48,0.09) | (0.83,0.15)/(0.40,0.10) |
| OTU22 | *Proteobacteria* | *Betaproteobacteria* | *Rhodocyclales* | *Rhodocyclaceae* |  | (1.61,0.16)/(0.45,0.10) | (1.99,0.23)/(0.41,0.09) | (1.67,0.17)/(0.24,0.05) | (1.90,0.15)/(0.38,0.09) |
| OTU26 | *Proteobacteria* | *Betaproteobacteria* | *Rhodocyclales* | *Rhodocyclaceae* |  | (1.30,0.08)/(2.28,0.43) | (0.40,0.06)/(0.75,0.17) | (0.34,0.04)/(0.24,0.09) | (0.17,0.09)/(0.19,0.15) |
| OTU27 | *Proteobacteria* | *Betaproteobacteria* | *Burkholderiales* | *Comamonadaceae* | *Alicycliphilus* | (0.35,0.07)/(0.17,0.08) | (0.47,0.25)/(2.47,2.34) | (0.45,0.10)/(0.41,0.17) | (0.51,0.21)/(0.72,0.20) |
| OTU2 | *Proteobacteria* | *Deltaproteobacteria* | *Syntrophobacterales* | *Syntrophobacteraceae* |  | (5.53,0.23)/(14.75,3.52) | (12.86,1.21)/(13.61,2.21) | (5.39,0.58)/(10.01,0.40) | (9.07,0.52)/(12.52,0.84) |
| OTU3 | *Proteobacteria* | *Deltaproteobacteria* | *Desulfobacterales* | *Desulfobacteraceae* | *Desulfobacterium* | (2.97,0.44)/(10.61,9.03) | (1.64,1.43)/(6.00,5.21) | (1.30,0.17)/(6.12,0.92) | (0.92,0.78)/(3.26,2.82) |
| OTU17 | *Proteobacteria* | *Deltaproteobacteria* | *Syntrophobacterales* | *Syntrophaceae* | *Desulfomonile* | (0.79,0.02)/(1.40,0.38) | (0.50,0.15)/(1.78,0.90) | (0.33,0.03)/(1.13,0.42) | (0.35,0.13)/(0.78,0.17) |
| OTU30 | *Proteobacteria* | *Deltaproteobacteria* | *Syntrophobacterales* | *Syntrophaceae* | *Desulfomonile* | (1.17,0.20)/(0.93,0.53) | (0.90,0.33)/(1.05,0.48) | (0.52,0.05)/(0.53,0.29) | (0.62,0.11)/(0.47,0.14) |
| OTU28 | *Proteobacteria* | *Deltaproteobacteria* | *Desulfobacterales* | *Desulfobacteraceae* |  | (1.01,0.08)/(0.53,0.22) | (0.96,0.20)/(0.53,0.08) | (0.60,0.09)/(0.26,0.10) | (0.63,0.15)/(0.53,0.13) |
| OTU43 | *Proteobacteria* | *Deltaproteobacteria* | *Desulfovibrionales* | *Desulfomicrobiaceae* | *Desulfomicrobium* | (1.13,0.07)/(0.52,0.14) | (1.02,0.23)/(0.19,0.09) | (0.61,0.13)/(0.16,0.09) | (0.51,0.12)/(0.16,0.08) |
| OTU25 | *Proteobacteria* | *Gammaproteobacteria* | *Pseudomonadales* | *Pseudomonadaceae* |  | (0.30,0.10)/(1.64,0.75) | (0.82,1.02)/(1.77,2.09) | (0.13,0.05)/(0.37,0.03) | (0.08,0.05)/(0.14,0.08) |
| OTU11 | *Bacteroidetes* |  |  |  |  | (4.23,0.25)/(0.53,0.09) | (2.48,0.63)/(0.98,0.19) | (2.33,0.31)/(0.73,0.35) | (1.88,0.21)/(1.17,0.24) |
| OTU12 | *Bacteroidetes* |  |  |  |  | (3.90,0.06)/(0.04,0.03) | (3.15,0.23)/(0.08,0.02) | (2.34,0.29)/(0.05,0.05) | (2.44,0.39)/(0.20,0.01) |
| OTU13 | *Bacteroidetes* |  |  |  |  | (2.41,0.14)/(0.13,0.03) | (1.93,0.34)/(0.21,0.08) | (1.70,0.11)/(0.29,0.19) | (2.20,0.31)/(0.73,0.33) |
| OTU36 | *Bacteroidetes* | *Flavobacteriia* | *Flavobacteriales* | *Flavobacteriaceae* | *Flavobacterium* | (0.36,0.05)/(0.02,0.02) | (0.36,0.10)/(0.10,0.05) | (2.86,0.21)/(0.20,0.10) | (1.81,0.49)/(0.23,0.03) |
| OTU14 | *Bacteroidetes* | *Sphingobacteriia* | *Sphingobacteriales* | *Chitinophagaceae* | *Sediminibacterium* | (0.07,0.05)/(0.01,0.02) | (0.08,0.06)/(0.22,0.33) | (2.98,1.64)/(0.24,0.19) | (1.47,1.55)/(0.34,0.21) |
| OTU21 | *Bacteroidetes* |  |  |  |  | (0.66,0.05)/(0.12,0.09) | (0.70,0.27)/(0.29,0.10) | (0.96,0.30)/(0.31,0.26) | (1.36,0.67)/(0.54,0.14) |
| OTU32 | *Bacteroidetes* |  |  |  |  | (0.01,0.00)/(0.03,0.02) | (0.33,0.28)/(0.31,0.16) | (1.69,0.43)/(0.78,0.14) | (0.92,0.11)/(0.19,0.10) |
| OTU6 | *Actinobacteria* | *Actinobacteria* | *Actinomycetales* | *Nocardiaceae* | *Rhodococcus* | (0.48,0.23)/(20.83,7.15) | (0.54,0.06)/(8.21,1.60) | (0.10,0.07)/(5.63,1.06) | (0.20,0.13)/(5.76,0.19) |
| OTU8 | *Chloroflexi* | *Anaerolineae* | *Anaerolineales* | *Anaerolineaceae* |  | (6.17,0.15)/(1.60,0.42) | (5.47,0.58)/(1.19,0.23) | (3.46,0.31)/(1.30,0.54) | (4.15,0.20)/(1.67,0.56) |
| OTU9 | *Chloroflexi* | *Anaerolineae* | *Anaerolineales* | *Anaerolineaceae* |  | (1.83,0.10)/(1.98,0.41) | (2.20,0.41)/(2.03,0.59) | (1.93,0.27)/(2.62,1.24) | (2.16,0.14)/(3.26,1.43) |

**Supplementary Table S2.**

**The 33 significantly enriched OTUs in the quinoline-degrading nitrate-reducing cultures, identified using H0 sample as a baseline by LEfSe.**

| **OTU ID** | **Taxonomical assignments** | | | | | **Riched time point** | **Relative abundance (%)**  **(mean, SD)** | | | |
| --- | --- | --- | --- | --- | --- | --- | --- | --- | --- | --- |
|  | Phylum | Class | Order | Family | Genus |  | H0 | H25 | H80 | H144 |
| OTU1 | *Proteobacteria* | *Betaproteobacteria* | *Rhodocyclales* | *Rhodocyclaceae* | *Thauera* | H25,H80,H144 | 12.17,0.07 | 19.89,0.14 | 38.78,0.24 | 30.68,0.31 |
| OTU31 | *Proteobacteria* | *Alphaproteobacteria* | *Rhizobiales* | *Rhizobiaceae* | *Rhizobium* | H25,H80,H144 | 0.12,0.00 | 0.46,0.28 | 0.45,0.43 | 0.85,0.11 |
| OTU32 | *Bacteroidetes* |  |  |  |  | H25,H80,H144 | 0.01,0.04 | 0.33,0.05 | 1.69,0.02 | 0.92,0.13 |
| OTU49 | *Chloroflexi* | *Anaerolineae* | *Anaerolineales* | *Anaerolineaceae* | *Anaerolinea* | H25,H80,H144 | 0.09,0.00 | 0.27,0.05 | 0.15,0.01 | 0.32,0.01 |
| OTU139 | *Proteobacteria* | *Betaproteobacteria* | *Burkholderiales* | *Comamonadaceae* | *Acidovorax* | H25,H80 | 0.04,0.01 | 0.09,0.08 | 0.09,0.07 | 0.01,0.15 |
| OTU24 | *Proteobacteria* | *Betaproteobacteria* | *Burkholderiales* |  |  | H25,H80 | 0.08,0.23 | 0.18,1.21 | 0.30,0.58 | 0.18,0.52 |
| OTU2 | *Proteobacteria* | *Deltaproteobacteria* | *Syntrophobacterales* | *Syntrophobacteraceae* |  | H25,H144 | 5.53,0.05 | 12.86,0.07 | 5.39,0.06 | 9.07,0.07 |
| OTU41 | *Chloroflexi* | *Anaerolineae* | *Anaerolineales* | *Anaerolineaceae* |  | H80,H144 | 0.22,0.07 | 0.21,0.06 | 0.35,0.13 | 0.39,0.12 |
| OTU38 | *Proteobacteria* | *Betaproteobacteria* | *Burkholderiales* | *Comamonadaceae* | *Simplicispira* | H80,H144 | 0.11,0.05 | 0.16,0.10 | 0.40,0.21 | 0.33,0.49 |
| OTU36 | *Bacteroidetes* | *Flavobacteriia* | *Flavobacteriales* | *Flavobacteriaceae* | *Flavobacterium* | H80,H144 | 0.36,0.05 | 0.36,0.06 | 2.86,1.64 | 1.81,1.55 |
| OTU14 | *Bacteroidetes* | *Sphingobacteriia* | *Sphingobacteriales* | *Chitinophagaceae* | *Sediminibacterium* | H80,H144 | 0.07,0.01 | 0.08,0.03 | 2.98,0.25 | 1.47,0.55 |
| OTU45 | *Bacteroidetes* | *Sphingobacteriia* | *Sphingobacteriales* | *Chitinophagaceae* |  | H80,H144 | 0.01,0.01 | 0.03,0.01 | 0.66,0.02 | 0.57,0.06 |
| OTU56 | Bacteria |  |  |  |  | H80,H144 | 0.01,0.01 | 0.00,0.03 | 0.12,0.01 | 0.92,0.02 |
| OTU144 | *Proteobacteria* | *Alphaproteobacteria* | *Rhizobiales* |  |  | H25 | 0.00,0.02 | 0.06,0.09 | 0.01,0.03 | 0.01,0.06 |
| OTU98 | *Chloroflexi* | *Anaerolineae* | *Anaerolineales* | *Anaerolineaceae* |  | H25 | 0.04,0.02 | 0.18,0.06 | 0.04,0.03 | 0.07,0.08 |
| OTU93 | *Chloroflexi* | *Anaerolineae* | *Anaerolineales* | *Anaerolineaceae* |  | H25 | 0.10,0.08 | 0.19,0.04 | 0.03,0.06 | 0.21,0.05 |
| OTU46 | *Chloroflexi* | *Anaerolineae* | *Anaerolineales* | *Anaerolineaceae* |  | H25 | 0.32,0.02 | 0.40,0.05 | 0.20,0.01 | 0.35,0.01 |
| OTU128 | *Proteobacteria* | *Betaproteobacteria* | *Hydrogenophilales* | *Hydrogenophilaceae* | *Thiobacillus* | H25 | 0.04,0.01 | 0.11,0.02 | 0.04,0.01 | 0.05,0.01 |
| OTU176 | *Proteobacteria* | *Gammaproteobacteria* | *Xanthomonadales* | *Xanthomonadaceae* | *Pseudoxanthomonas* | H25 | 0.02,0.06 | 0.06,0.01 | 0.01,0.07 | 0.00,0.03 |
| OTU96 | Bacteria |  |  |  |  | H25 | 0.11,0.00 | 0.19,0.00 | 0.14,0.02 | 0.09,0.10 |
| OTU146 | *Bacteroidetes* | *Sphingobacteriia* | *Sphingobacteriales* | *Chitinophagaceae* | *Terrimonas* | H80 | 0.00,0.05 | 0.00,0.27 | 0.08,0.30 | 0.07,0.67 |
| OTU21 | *Bacteroidetes* |  |  |  |  | H80 | 0.66,0.02 | 0.70,0.03 | 0.96,0.03 | 1.36,0.06 |
| OTU121 | *Chloroflexi* | *Anaerolineae* | *Anaerolineales* | *Anaerolineaceae* | *Levilinea* | H80 | 0.08,0.00 | 0.11,0.01 | 0.11,0.06 | 0.13,0.02 |
| OTU64 | *Verrucomicrobia* | *Opitutae* | *Opitutales* | *Opitutaceae* |  | H80 | 0.00,0.01 | 0.01,0.02 | 0.09,0.01 | 0.02,0.00 |
| OTU85 | Bacteria |  |  |  |  | H80 | 0.10,0.04 | 0.07,0.01 | 0.12,0.03 | 0.00,0.18 |
| OTU141 | *Bacteroidetes* |  |  |  |  | H144 | 0.07,0.01 | 0.05,0.02 | 0.12,0.03 | 0.68,0.08 |
| OTU106 | *Chloroflexi* | *Anaerolineae* | *Anaerolineales* | *Anaerolineaceae* |  | H144 | 0.02,0.10 | 0.01,0.41 | 0.02,0.27 | 0.10,0.14 |
| OTU9 | *Chloroflexi* | *Anaerolineae* | *Anaerolineales* | *Anaerolineaceae* |  | H144 | 1.83,0.01 | 2.20,0.01 | 1.93,0.02 | 2.16,0.04 |
| OTU224 | *Firmicutes* | *Clostridia* | *Clostridiales* | *Clostridiales_Incertae Sedis XIII* | *Anaerovorax* | H144 | 0.03,0.02 | 0.00,0.00 | 0.03,0.08 | 0.10,0.01 |
| OTU113 | *Ignavibacteriae* | *Ignavibacteria* | *Ignavibacteriales* | *Ignavibacteriaceae* | *Ignavibacterium* | H144 | 0.01,0.08 | 0.00,0.93 | 0.05,0.39 | 0.08,1.05 |
| OTU5 | *Proteobacteria* | *Betaproteobacteria* | *Rhodocyclales* | *Rhodocyclaceae* |  | H144 | 4.32,0.01 | 5.03,0.03 | 4.64,0.00 | 5.65,0.27 |
| OTU34 | Bacteria |  |  |  |  | H144 | 0.00,0.01 | 0.03,0.00 | 0.01,0.00 | 0.36,0.16 |
| OTU73 | Bacteria |  |  |  |  | H144 | 0.00,0.00 | 0.00,0.00 | 0.00,0.00 | 1.15,0.00 |

**Supplementary Table S3 50 significant active OTUs identified by comparing the DNA and RNA sample at H0, H25, H80 and H144 by LEfSe.**

| **OTU ID** | **Taxonomical assignments** | | | | | **Actived group** | **Relative abundance (%)**  **Mean(DNA),SD/Mean(RNA),SD** | | | |
| --- | --- | --- | --- | --- | --- | --- | --- | --- | --- | --- |
|  | **Phylum** | **Class** | **Order** | **Family** | **Genus** |  | **H0** | **H25** | **H80** | **H144** |
| OTU7 | *Proteobacteria* | *Betaproteobacteria* | *Rhodocyclales* | *Rhodocyclaceae* |  | H0,H25,H80,H144 | (3.20,0.25)/(3.88,0.21) | (1.87,0.19)/(3.02,0.16) | (1.29,0.03)/(1.43,0.03) | (1.15,0.04)/(1.98,0.12) |
| OTU17 | *Proteobacteria* | *Deltaproteobacteria* | *Syntrophobacterales* | *Syntrophaceae* | *Desulfomonile* | H0,H25,H80,H144 | (0.79,0.02)/(1.40,0.38) | (0.50,0.15)/(1.78,0.90) | (0.33,0.03)/(1.13,0.42) | (0.35,0.13)/(0.78,0.17) |
| OTU53 | *Lentisphaerae* | *Oligosphaeria* | *Oligosphaerales* | *Oligosphaeraceae* | *Oligosphaera* | H0,H25,H80,H144 | (0.08,0.03)/(0.67,0.48) | (0.08,0.05)/(0.22,0.06) | (0.03,0.03)/(0.39,0.18) | (0.10,0.04)/(0.33,0.06) |
| OTU59 | *Proteobacteria* | *Alphaproteobacteria* | *Rhizobiales* | *Methylocystaceae* | *Methylocystis* | H0,H25,H80,H144 | (0.01,0.02)/(0.68,0.43) | (0.02,0.01)/(0.55,0.05) | (0.00,0.00)/(0.30,0.01) | (0.02,0.02)/(0.19,0.08) |
| OTU6 | *Actinobacteria* | *Actinobacteria* | *Actinomycetales* | *Nocardiaceae* | *Rhodococcus* | H0,H25,H80,H144 | (0.48,0.23)/(20.83,7.15) | (0.54,0.06)/(8.21,1.60) | (0.10,0.07)/(5.63,1.06) | (0.20,0.13)/(5.76,0.19) |
| OTU148 | Unclassified |  |  |  |  | H0,H25,H80,H144 | (0.01,0.01)/(0.09,0.01) | (0.00,0.01)/(0.12,0.10) | (0.01,0.01)/(0.08,0.05) | (0.01,0.01)/(0.12,0.02) |
| OTU82 | Unclassified |  |  |  |  | H0,H25,H80,H144 | (0.01,0.01)/(0.21,0.10) | (0.02,0.01)/(0.15,0.10) | (0.01,0.01)/(0.20,0.09) | (0.01,0.01)/(0.15,0.15) |
| OTU2 | *Proteobacteria* | *Deltaproteobacteria* | *Syntrophobacterales* | *Syntrophobacteraceae* |  | H0,H80,H144 | (5.53,0.23)/(14.75,3.52) | (12.86,1.21)/(13.61,2.21) | (5.39,0.58)/(10.01,0.40) | (9.07,0.52)/(12.52,0.84) |
| OTU10 | Unclassified |  |  |  |  | H0,H80,H144 | (0.98,0.23)/(4.29,1.38) | (1.20,0.30)/(2.98,1.71) | (0.65,0.06)/(2.23,0.51) | (0.71,0.24)/(1.64,0.20) |
| OTU34 | Unclassified |  |  |  |  | H0,H80,H144 | (0.00,0.01)/(0.29,0.24) | (0.03,0.03)/(0.13,0.14) | (0.01,0.00)/(0.10,0.03) | (0.36,0.27)/(4.07,3.76) |
| OTU119 | *Hydrogenedentes* | *Candidatus Hydrogenedens* |  |  |  | H0,H25,H80 | (0.03,0.02)/(0.24,0.10) | (0.01,0.01)/(0.11,0.05) | (0.00,0.01)/(0.05,0.02) | (0.00,0.00)/(0.03,0.03) |
| OTU125 | Unclassified |  |  |  |  | H0,H25,H144 | (0.01,0.02)/(0.23,0.07) | (0.00,0.01)/(0.09,0.05) | (0.00,0.00)/(0.01,0.01) | (0.00,0.01)/(0.06,0.04) |
| OTU20 | *Acidobacteria* | *Gp7* |  |  |  | H25,H80,H144 | (0.56,0.20)/(0.22,0.13) | (0.26,0.05)/(1.69,0.47) | (0.21,0.05)/(0.70,0.22) | (0.20,0.06)/(1.31,0.52) |
| OTU76 | *Proteobacteria* | *Alphaproteobacteria* | *Rhodospirillales* | *Rhodospirillaceae* | *Magnetospirillum* | H25,H80,H144 | (0.17,0.02)/(0.09,0.01) | (0.04,0.03)/(0.15,0.04) | (0.04,0.01)/(0.22,0.03) | (0.02,0.02)/(0.16,0.09) |
| OTU1 | *Proteobacteria* | *Betaproteobacteria* | *Rhodocyclales* | *Rhodocyclaceae* | *Thauera* | H25,H80 | (12.17,0.87)/(10.36,4.07) | (19.89,4.66)/(29.07,3.56) | (38.78,2.67)/(50.10,3.74) | (30.68,2.50)/(37.92,5.76) |
| OTU164 | *Proteobacteria* | *Betaproteobacteria* | *Burkholderiales* | *Comamonadaceae* |  | H25,H80 | (0.00,0.01)/(0.01,0.02) | (0.01,0.01)/(0.05,0.02) | (0.01,0.01)/(0.04,0.03) | (0.00,0.01)/(0.04,0.04) |
| OTU78 | *Proteobacteria* | *Betaproteobacteria* | *Burkholderiales* | *Comamonadaceae* |  | H25,H80 | (0.07,0.04)/(0.00,0.00) | (0.04,0.00)/(0.12,0.06) | (0.11,0.04)/(0.31,0.09) | (0.16,0.06)/(0.07,0.04) |
| OTU166 | Unclassified |  |  |  |  | H25,H144 | (0.01,0.01)/(0.01,0.01) | (0.01,0.01)/(0.04,0.02) | (0.00,0.00)/(0.01,0.01) | (0.00,0.01)/(0.10,0.07) |
| OTU140 | Unclassified |  |  |  |  | H25,H144 | (0.05,0.03)/(0.05,0.04) | (0.00,0.00)/(0.05,0.04) | (0.01,0.01)/(0.01,0.01) | (0.00,0.00)/(0.03,0.01) |
| OTU64 | *Verrucomicrobia* | *Opitutae* | *Opitutales* | *Opitutaceae* |  | H80,H144 | (0.00,0.00)/(0.02,0.01) | (0.01,0.01)/(0.17,0.22) | (0.09,0.06)/(0.19,0.02) | (0.02,0.02)/(0.09,0.05) |
| OTU24 | *Proteobacteria* | *Betaproteobacteria* | *Burkholderiales* |  |  | H80,H144 | (0.08,0.01)/(0.08,0.04) | (0.18,0.08)/(0.27,0.04) | (0.30,0.07)/(1.33,0.30) | (0.18,0.15)/(1.41,0.25) |
| OTU26 | *Proteobacteria* | *Betaproteobacteria* | *Rhodocyclales* | *Rhodocyclaceae* |  | H0,H25 | (1.30,0.08)/(2.28,0.43) | (0.40,0.06)/(0.75,0.17) | (0.34,0.04)/(0.24,0.09) | (0.17,0.09)/(0.19,0.15) |
| OTU228 | *Firmicutes* | *Clostridia* | *Clostridiales* | *Clostridiaceae 1* | *Clostridium sensu stricto* | H0,H25 | (0.00,0.00)/(0.03,0.02) | (0.00,0.01)/(0.03,0.02) | (0.00,0.00)/(0.01,0.02) | (0.00,0.00)/(0.01,0.01) |
| OTU156 | Unclassified |  |  |  |  | H0,H25 | (0.02,0.01)/(0.10,0.04) | (0.01,0.01)/(0.03,0.02) | (0.02,0.00)/(0.00,0.01) | (0.02,0.02)/(0.02,0.02) |
| OTU152 | Unclassified |  |  |  |  | H0,H25 | (0.00,0.00)/(0.08,0.02) | (0.00,0.01)/(0.06,0.04) | (0.01,0.01)/(0.01,0.01) | (0.00,0.00)/(0.00,0.01) |
| OTU182 | *Proteobacteria* | *Deltaproteobacteria* | *Myxococcales* |  |  | H0,H25 | (0.01,0.01)/(0.07,0.05) | (0.00,0.01)/(0.12,0.13) | (0.01,0.02)/(0.01,0.02) | (0.00,0.01)/(0.00,0.00) |
| OTU225 | *Proteobacteria* | *Gammaproteobacteria* | *Gammaproteobacteria_incertae_sedis* | |  | H0 | (0.01,0.00)/(0.04,0.00) | (0.01,0.02)/(0.02,0.03) | (0.02,0.02)/(0.02,0.03) | (0.00,0.00)/(0.02,0.02) |
| OTU226 | *Proteobacteria* |  |  |  |  | H0 | (0.01,0.01)/(0.12,0.09) | (0.00,0.01)/(0.00,0.00) | (0.00,0.00)/(0.02,0.02) | (0.00,0.00)/(0.00,0.00) |
| OTU111 | *Verrucomicrobia* | *Opitutae* | *Opitutales* | *Opitutaceae* | *Opitutus* | H25 | (0.07,0.02)/(0.04,0.02) | (0.07,0.03)/(0.13,0.04) | (0.05,0.01)/(0.06,0.02) | (0.08,0.06)/(0.03,0.03) |
| OTU126 | Unclassified |  |  |  |  | H25 | (0.04,0.02)/(0.03,0.02) | (0.04,0.01)/(0.06,0.00) | (0.02,0.03)/(0.03,0.04) | (0.04,0.04)/(0.05,0.04) |
| OTU132 | Unclassified |  |  |  |  | H25 | (0.04,0.01)/(0.05,0.04) | (0.01,0.01)/(0.04,0.02) | (0.02,0.01)/(0.03,0.03) | (0.02,0.02)/(0.01,0.02) |
| OTU153 | *Proteobacteria* | *Epsilonproteobacteria* | *Campylobacterales* |  |  | H25 | (0.11,0.09)/(0.00,0.01) | (0.00,0.01)/(0.03,0.02) | (0.02,0.01)/(0.01,0.02) | (0.02,0.02)/(0.01,0.01) |
| OTU195 | Unclassified |  |  |  |  | H25 | (0.01,0.01)/(0.03,0.04) | (0.01,0.02)/(0.14,0.10) | (0.04,0.02)/(0.02,0.02) | (0.02,0.01)/(0.00,0.01) |
| OTU27 | *Proteobacteria* | *Betaproteobacteria* | *Burkholderiales* | *Comamonadaceae* | *Alicycliphilus* | H25 | (0.35,0.07)/(0.17,0.08) | (0.47,0.25)/(2.47,2.34) | (0.45,0.10)/(0.41,0.17) | (0.51,0.21)/(0.72,0.20) |
| OTU40 | *Proteobacteria* | *Betaproteobacteria* | *Rhodocyclales* | *Rhodocyclaceae* |  | H25 | (0.94,0.17)/(1.22,0.29) | (0.25,0.09)/(0.47,0.06) | (0.18,0.06)/(0.14,0.07) | (0.02,0.00)/(0.03,0.03) |
| OTU51 | *Proteobacteria* | *Alphaproteobacteria* | *Rhizobiales* | *Hyphomicrobiaceae* | *Hyphomicrobium* | H25 | (0.11,0.04)/(0.09,0.06) | (0.20,0.08)/(0.40,0.18) | (0.10,0.05)/(0.17,0.07) | (0.12,0.05)/(0.17,0.05) |
| OTU127 | *Planctomycetes* | *Planctomycetia* | *Planctomycetales* | *Planctomycetaceae* |  | H80 | (0.04,0.04)/(0.04,0.04) | (0.03,0.03)/(0.08,0.04) | (0.00,0.00)/(0.04,0.01) | (0.01,0.01)/(0.03,0.01) |
| OTU154 | *Chloroflexi* | *Anaerolineae* | *Anaerolineales* | *Anaerolineaceae* |  | H80 | (0.03,0.00)/(0.02,0.02) | (0.00,0.00)/(0.00,0.00) | (0.01,0.00)/(0.04,0.04) | (0.02,0.01)/(0.06,0.10) |
| OTU39 | *Chloroflexi* | *Anaerolineae* | *Anaerolineales* | *Anaerolineaceae* |  | H80 | (0.44,0.21)/(0.48,0.03) | (0.28,0.04)/(0.33,0.14) | (0.24,0.06)/(0.49,0.02) | (0.38,0.17)/(0.64,0.19) |
| OTU3 | *Proteobacteria* | *Deltaproteobacteria* | *Desulfobacterales* | *Desulfobacteraceae* | *Desulfobacterium* | H80 | (2.97,0.44)/(10.61,9.03) | (1.64,1.43)/(6.00,5.21) | (1.30,0.17)/(6.12,0.92) | (0.92,0.78)/(3.26,2.82) |
| OTU74 | *Hydrogenedentes* | *Candidatus Hydrogenedens* |  |  |  | H80 | (0.18,0.09)/(0.22,0.21) | (0.05,0.05)/(0.13,0.12) | (0.04,0.00)/(0.15,0.05) | (0.01,0.01)/(0.06,0.05) |
| OTU83 | Unclassified |  |  |  |  | H80 | (0.07,0.03)/(0.28,0.37) | (0.04,0.05)/(0.10,0.10) | (0.03,0.01)/(0.09,0.02) | (0.04,0.04)/(0.04,0.03) |
| OTU25 | *Proteobacteria* | *Gammaproteobacteria* | *Pseudomonadales* | *Pseudomonadaceae* |  | H0,H80 | (0.30,0.10)/(1.64,0.75) | (0.82,1.02)/(1.77,2.09) | (0.13,0.05)/(0.37,0.03) | (0.08,0.05)/(0.14,0.08) |
| OTU47 | Unclassified |  |  |  |  | H0,H80 | (0.22,0.06)/(0.49,0.17) | (0.28,0.07)/(0.32,0.05) | (0.11,0.04)/(0.32,0.04) | (0.31,0.04)/(0.30,0.11) |
| OTU173 | Unclassified |  |  |  |  | H0,H80 | (0.00,0.01)/(0.05,0.03) | (0.02,0.02)/(0.05,0.03) | (0.00,0.00)/(0.03,0.02) | (0.01,0.01)/(0.01,0.02) |
| OTU55 | *Lentisphaerae* | *Lentisphaeria* | *Victivallales* | *Victivallaceae* | *Victivallis* | H144 | (0.09,0.04)/(0.03,0.04) | (0.00,0.00)/(0.03,0.02) | (0.02,0.02)/(0.03,0.02) | (0.19,0.07)/(1.16,0.11) |
| OTU9 | *Chloroflexi* | *Anaerolineae* | *Anaerolineales* | *Anaerolineaceae* |  | H144 | (1.83,0.10)/(1.98,0.41) | (2.20,0.41)/(2.03,0.59) | (1.93,0.27)/(2.62,1.24) | (2.16,0.14)/(3.26,1.43) |
| OTU90 | *Chloroflexi* |  |  |  |  | H144 | (0.14,0.03)/(0.14,0.19) | (0.11,0.04)/(0.11,0.06) | (0.06,0.03)/(0.10,0.02) | (0.09,0.07)/(0.24,0.01) |
| OTU147 | Unclassified |  |  |  |  | H144 | (0.02,0.02)/(0.02,0.01) | (0.03,0.01)/(0.03,0.03) | (0.03,0.03)/(0.05,0.02) | (0.02,0.02)/(0.05,0.02) |
| OTU144 | *Proteobacteria* | *Alphaproteobacteria* | *Rhizobiales* |  |  | H144 | (0.00,0.01)/(0.03,0.03) | (0.06,0.03)/(0.04,0.05) | (0.01,0.01)/(0.04,0.06) | (0.01,0.02)/(0.07,0.04) |

**Supplementary Table S4**

**Thirty OTUs that were significantly correlated with physiological parameters during quinoline degradation.** The associations(R) between the OTUs abundances and physiological parameters assessed by the Spearman’s correlations. P values were adjusted as described by Benjamini and Hochberg. (false discovery ratio<0.05).

| **OTU ID** | **CAG group** | **Taxonmical assignments** | | | | | **R-Value and P-value** | | | | | |
| --- | --- | --- | --- | --- | --- | --- | --- | --- | --- | --- | --- | --- |
|  |  | **phylum** | **class** | **order** | **family** | **genus** | **Quinoline** | **Ammonia** | **Nitrate** | **Nitrite** | **Nitrogen** | **Mehtane** |
| OTU32 | CAG9 | *Bacteroidetes* |  |  |  |  | (-0.67,0.0005) | (0.86,0.0000) | (-0.81,0.0000) | (-0.07,0.7469) | (0.67,0.0005) | (0.17,0.5231) |
| OTU146 | CAG9 | *Bacteroidetes* | *Sphingobacteriia* | *Sphingobacteriales* | *Chitinophagaceae* | *Terrimonas* | (-0.49,0.0235) | (0.56,0.0153) | (-0.55,0.0153) | (-0.24,0.2936) | (0.49,0.0235) | (0.22,0.2936) |
| OTU14 | CAG9 | *Bacteroidetes* | *Sphingobacteriia* | *Sphingobacteriales* | *Chitinophagaceae* | *Sediminibacterium* | (-0.71,0.0002) | (0.72,0.0002) | (-0.75,0.0001) | (-0.28,0.1880) | (0.71,0.0002) | (0.40,0.0607) |
| OTU45 | CAG10 | *Bacteroidetes* | *Sphingobacteriia* | *Sphingobacteriales* | *Chitinophagaceae* |  | (-0.83,0.0000) | (0.83,0.0000) | (-0.88,0.0000) | (-0.35,0.0891) | (0.83,0.0000) | (0.50,0.0153) |
| OTU56 | CAG10 | un_Bacteria |  |  |  |  | (-0.89,0.0000) | (0.70,0.0002) | (-0.84,0.0000) | (-0.47,0.0207) | (0.89,0.0000) | (0.76,0.0000) |
| OTU141 | CAG13 | *Bacteroidetes* |  |  |  |  | (-0.64,0.0015) | (0.28,0.1817) | (-0.49,0.0238) | (-0.34,0.1213) | (0.64,0.0015) | (0.77,0.0001) |
| OTU224 | CAG13 | *Firmicutes* | *Clostridia* | *Clostridiales* | *Clostridiales_Incertae Sedis XIII* | *Anaerovorax* | (-0.46,0.0363) | (0.17,0.4275) | (-0.33,0.1367) | (-0.47,0.0363) | (0.46,0.0363) | (0.63,0.0060) |
| OTU41 | CAG11 | *Chloroflexi* | *Anaerolineae* | *Anaerolineales* | *Anaerolineaceae* |  | (-0.81,0.0000) | (0.68,0.0003) | (-0.79,0.0000) | (-0.47,0.0197) | (0.81,0.0000) | (0.64,0.0009) |
| OTU78 | CAG11 | *Proteobacteria* | *Betaproteobacteria* | *Burkholderiales* | *Comamonadaceae* |  | (-0.54,0.0099) | (0.67,0.0019) | (-0.64,0.0023) | (-0.14,0.5167) | (0.54,0.0099) | (0.17,0.5167) |
| OTU113 | CAG8 | *Ignavibacteriae* | *Ignavibacteria* | *Ignavibacteriales* | *Ignavibacteriaceae* | *Ignavibacterium* | (-0.73,0.0001) | (0.52,0.0108) | (-0.66,0.0006) | (-0.40,0.0551) | (0.73,0.0001) | (0.68,0.0005) |
| OTU55 | CAG8 | *Lentisphaerae* | *Lentisphaeria* | *Victivallales* | *Victivallaceae* | *Victivallis* | (-0.47,0.0323) | (0.08,0.7148) | (-0.29,0.2080) | (-0.50,0.0323) | (0.47,0.0323) | (0.74,0.0002) |
| OTU73 | CAG8 | un_Bacteria |  |  |  |  | (-0.66,0.0008) | (0.16,0.4527) | (-0.43,0.0508) | (-0.36,0.0971) | (0.66,0.0008) | (0.93,0.0000) |
| OTU1 | CAG6 | *Proteobacteria* | *Betaproteobacteria* | *Rhodocyclales* | *Rhodocyclaceae* | *Thauera* | (-0.76,0.0000) | (0.93,0.0000) | (-0.89,0.0000) | (-0.21,0.3282) | (0.76,0.0000) | (0.26,0.2548) |
| OTU24 | CAG6 | *Proteobacteria* | *Betaproteobacteria* | *Burkholderiales* |  |  | (-0.63,0.0015) | (0.71,0.0003) | (-0.71,0.0003) | (-0.04,0.8466) | (0.63,0.0015) | (0.25,0.2860) |
| OTU64 | CAG6 | *Verrucomicrobia* | *Opitutae* | *Opitutales* | *Opitutaceae* |  | (-0.49,0.0224) | (0.70,0.0009) | (-0.63,0.0032) | (-0.08,0.7939) | (0.49,0.0224) | (0.06,0.7939) |
| OTU31 | CAG9 | *Proteobacteria* | *Alphaproteobacteria* | *Rhizobiales* | *Rhizobiaceae* | *Rhizobium* | (-0.62,0.0033) | (0.48,0.0268) | (-0.58,0.0057) | (0.14,0.5171) | (0.62,0.0033) | (0.44,0.0353) |
| OTU38 | CAG9 | *Proteobacteria* | *Betaproteobacteria* | *Burkholderiales* | *Comamonadaceae* | *Simplicispira* | (-0.50,0.0207) | (0.64,0.0044) | (-0.60,0.0060) | (-0.03,0.8974) | (0.50,0.0207) | (0.11,0.7258) |
| OTU34 | CAG5 | un_Bacteria |  |  |  |  | (-0.50,0.0252) | (0.14,0.5138) | (-0.34,0.1594) | (-0.18,0.4772) | (0.50,0.0252) | (0.67,0.0022) |
| OTU51 | CAG5 | *Proteobacteria* | *Alphaproteobacteria* | *Rhizobiales* | *Hyphomicrobiaceae* | *Hyphomicrobium* | (-0.07,0.9403) | (0.02,0.9403) | (-0.05,0.9403) | (0.60,0.0122) | (0.07,0.9403) | (-0.03,0.9403) |
| OTU119 | CAG2 | *Hydrogenedentes* | *Candidatus Hydrogenedens* |  |  |  | (0.50,0.0295) | (-0.43,0.0555) | (0.49,0.0295) | (0.07,0.7423) | (-0.50,0.0295) | (-0.35,0.1076) |
| OTU140 | CAG2 | un_Bacteria |  |  |  |  | (0.51,0.0174) | (-0.54,0.0174) | (0.55,0.0174) | (0.01,0.9736) | (-0.51,0.0174) | (-0.23,0.3394) |
| OTU7 | CAG2 | *Proteobacteria* | *Betaproteobacteria* | *Rhodocyclales* | *Rhodocyclaceae* |  | (0.79,0.0000) | (-0.82,0.0000) | (0.85,0.0000) | (0.18,0.3981) | (-0.79,0.0000) | (-0.40,0.0609) |
| OTU25 | CAG2 | *Proteobacteria* | *Gammaproteobacteria* | *Pseudomonadales* | *Pseudomonadaceae* |  | (0.70,0.0004) | (-0.51,0.0128) | (0.64,0.0012) | (0.42,0.0426) | (-0.70,0.0004) | (-0.64,0.0012) |
| OTU182 | CAG2 | *Proteobacteria* | *Deltaproteobacteria* | *Myxococcales* |  |  | (0.48,0.0437) | (-0.40,0.0745) | (0.47,0.0437) | (0.28,0.1845) | (-0.48,0.0437) | (-0.39,0.0745) |
| OTU26 | CAG4 | *Proteobacteria* | *Betaproteobacteria* | *Rhodocyclales* | *Rhodocyclaceae* |  | (0.90,0.0000) | (-0.78,0.0000) | (0.89,0.0000) | (0.19,0.3622) | (-0.90,0.0000) | (-0.63,0.0013) |
| OTU40 | CAG4 | *Proteobacteria* | *Betaproteobacteria* | *Rhodocyclales* | *Rhodocyclaceae* |  | (0.95,0.0000) | (-0.73,0.0001) | (0.89,0.0000) | (0.19,0.3622) | (-0.95,0.0000) | (-0.75,0.0000) |
| OTU226 | CAG4 | *Proteobacteria* |  |  |  |  | (0.55,0.0166) | (-0.39,0.0677) | (0.50,0.0270) | (-0.22,0.2936) | (-0.55,0.0166) | (-0.40,0.0677) |
| OTU154 | CAG4 | *Chloroflexi* | *Anaerolineae* | *Anaerolineales* | *Anaerolineaceae* |  | (0.11,0.9472) | (-0.06,0.9472) | (0.09,0.9472) | (-0.64,0.0043) | (-0.11,0.9472) | (0.01,0.9472) |
| OTU139 | CAG3 | *Proteobacteria* | *Betaproteobacteria* | *Burkholderiales* | *Comamonadaceae* | *Acidovorax* | (0.39,0.0858) | (-0.10,0.6340) | (0.26,0.2610) | (0.54,0.0185) | (-0.39,0.0858) | (-0.61,0.0089) |
| OTU85 | CAG3 | un_Bacteria |  |  |  |  | (0.46,0.0489) | (-0.08,0.7041) | (0.28,0.2670) | (0.11,0.7041) | (-0.46,0.0489) | (-0.65,0.0038) |
